# Complex Organic Matter Synthesis on Siloxyl Radicals in the Presence of CO

**DOI:** 10.3389/fchem.2020.621898

**Published:** 2021-02-01

**Authors:** Marco Fioroni, Nathan J. DeYonker

**Affiliations:** Department of Chemistry, The University of Memphis, Memphis, TN, United States

**Keywords:** astrochemistry, heterogeneous catalysis, silica surface, co polymerization, COM synthesis

## Abstract

Heterogeneous phase astrochemistry plays an important role in the synthesis of complex organic matter (COM) as found on comets and rocky body surfaces like asteroids, planetoids, moons and planets. The proposed catalytic model is based on two assumptions: **(a)** siliceous rocks in both crystalline or amorphous states show surface-exposed defective centers such as siloxyl (Si-O•) radicals; **(b)** the second phase is represented by gas phase CO molecules, an abundant C_1_ building block found in space. By means of quantum chemistry; (DFT, PW6B95/def2-TZVPP); the surface of a siliceous rock in presence of CO is modeled by a simple POSS (polyhedral silsesquioxane) where a siloxyl (Si-O•) radical is present. Four CO molecules have been consecutively added to the Si-O• radical and to the nascent polymeric CO (pCO) chain. The first CO insertion shows no activation free energy with ΔG_200*K*_ = −21.7 kcal/mol forming the SiO-CO• radical. The second and third CO insertions show Δ${\text{G}}_{200K}^{‡}$ ≤ 10.5 kcal/mol. Ring closure of the SiO-CO-CO• (oxalic anhydride) moiety as well as of the SiO-CO-CO-CO• system (di-cheto form of oxetane) are thermodynamically disfavored. The last CO insertion shows no free energy of activation resulting in the stable five member pCO ring, precursor to 1,4-epoxy-1,2,3-butanone. Hydrogenation reactions of the pCO have been considered on the SiO oxygen or on the carbons and oxygens of the pCO chains. The formation of the reactive aldehyde SiO-CHO on the siliceous surface is possible. In principle, the complete hydrogenation of the (CO)_1−4_ series results in the formation of methanol and polyols. Furthermore, all the SiO-pCO intermediates and the lactone 1,4-epoxy-1,2,3-butanone product in its radical form can be important building blocks in further polymerization reactions and/or open ring reactions with H (aldehydes, polyols) or CN (chetonitriles), resulting in highly reactive multi-functional compounds contributing to COM synthesis.

## 1. Introduction

The importance of heterogeneous phase catalysis in astrochemistry (solid to gas phase, solid to solid phase) is rapidly growing (Herbst, 2013; van Dishoeck, 2014; Cuppen et al., 2017).

In particular, dust grains play an important role in the chemistry of interstellar and circumstellar environments, where chemical reactions can occur on a large scale of molecular complexity starting from simple H_2_ to COM (Complex Organic Matter). A dust grain consists of a silicate core covered by an icy mantle, whose composition (H_2_O, CO, CO_2_, CH_4_, CH_3_OH, NH_3_) and ratio of the single constituents is a function of the astrophysical environment (Tielens, 2013; Boogert et al., 2015).

The silicate core composition is deduced by comparing astronomical IR observations with spectra from laboratory silicate samples, suggesting that Mg rich-silicates such as pyroxene (Mg_1−*n*_Fe_*n*_SiO_3_) and/or olivine (Mg_2−*n*_Fe_*n*_SiO_4_) in pure state or in mixtures are the main components (Jäger et al., 2003; Escatllar et al., 2019). Furthermore, the surface of the Mg-silicate core is probably covered by -OH groups (Kerkeni et al., 2017) marked by the presence of the 3.2 μm band detected by the Rosetta probe on the nucleus surface of comet 67P/ Churyumov–Gerasimenko (Mennella et al., 2020). However, due to the harsh conditions (T, radiation, collisions) and/or various reprocessing phases (Bromley et al., 2014), the silicate surface can be covered not only by silanols or charged groups but by siloxyl (SiO•) or silyl (Si•) radicals, too (Wang et al., 2018). The presence of radicals is a common phenomenon on silica surfaces in both the amorphous or crystalline states. In fact it is well-known that cleaved SiO_2_ surfaces are more reactive compared to the reconstructed ones, showing a relative higher surface energy due the presence of free valences on the Si and O atoms (Rignanese et al., 2000; Malyi et al., 2014).

Ideally the growth of a crystal or amorphous silica from single SiO units or the cleavage of a bulk of the same materials will expose a surface where siloxyl and silyl radicals are present. To satisfy the free valencies two main processes can be considered: **(a)** in case of a cleaved α-quartz (001) surface, an annealing at T≥400 K will partially or fully re-coordinate the Si and O atoms (Goumans et al., 2007); **(b)** molecules like water will cover the surface with silanol groups (SiOH), an important actor in the modulation of the physico-chemical characteristics of the crystalline/amorphous quartz surface (Comas-Vives, 2016; Schrader et al., 2018; Wang et al., 2018). As a consequence of their radical character, such surfaces will display a strong chemical reactivity. Recent studies on the carbonation and hydrolysis reactions on cleaved quartz, important for long term CO_2_ storage (Jia et al., 2019) as well as CO_2_ adsorption on cleaved α-quartz (001) (Malyi et al., 2015) also consider the presence of oxygen radicals on the surface.

**Figure 1:**
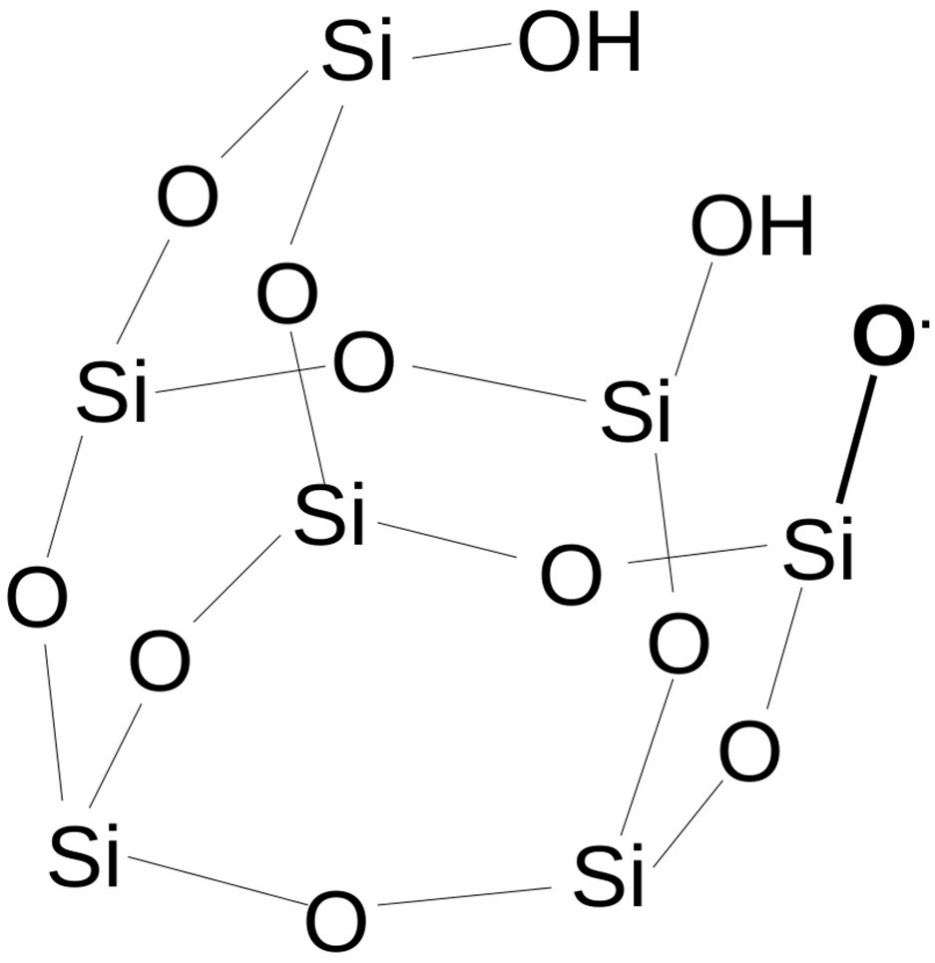
The siloxyl radical defect on the POSS model. Hydrogen atoms on Si atoms have been deleted for clarity.

Based on the previous assertions and shifting attention to cosmic dust grains, the following question can be formulated: *what kind of chemistry is developed at the interface between a radical center present on a siliceous rock and the gas*/*ice of molecular species of astrochemical interest like H*_2_*O or CO?* To answer the question, by means of quantum chemistry and by using a simplified model of an amorphous siliceous surface based on a silica-POSS (polyhedral-silsesquioxane) moiety (Fioroni et al., 2018) (Figure 1), a siloxyl radical (Si-O•) is modeled to react with CO molecules.

The POSS model used in our work is a partially condensed silsesquioxane [H_7_Si_7_O_9_(OH)_3_], where a single H atom is homolytically cleaved from one of the three *vicinal* OH groups to obtain a SiO• radical. Compared to a pyroxene or olivine silicate, POSS has no Mg atoms interacting with the silicate oxygens. Mg can play a role in the acid-base surface properties by the formation of magnesium-hydrate, and is certainly recognized as an important actor in astrochemical grain chemistry (Cornu et al., 2017). However, by only considering the siloxyl radical and because in a siliceous lattice the SiO_4_ or [SiO_2_]_*x*_ units and skeleton are present, the chemistry of a pure silica model will be reasonably transferable to a silicate when only the SiO chemistry is considered (Fioroni et al., 2018).

In the last 30 years, POSSs have given a fundamental contribution to understand surface reactions and catalysts activity on silica surfaces at the molecular level (Feher et al., 1989; Quadrelli and Basset, 2010). In particular the POSS ability to be a reliable model of a heterogeneous catalytic surface, allowed the use of a wide range of experimental as well as theoretical techniques to understand reaction mechanisms (Estes et al., 2016; Assefa et al., 2020).

The CO molecule is also of great astrochemical interest and is an abundant C_1_ building block that can develop complex organic chemistry under opportune (extreme P) conditions (Bernard et al., 1998; Lipp et al., 2005) or if catalyzed under normal conditions (West and Niu, 1970; Seitz and Imming, 1992). Before this work, studies on CO molecules interacting on a crystalline (001) α-quartz surface (Goumans et al., 2007) or an amorphous silica surface with siloxyl radicals (Goumans et al., 2008) were conducted, though no CO polymerization chemistry was analyzed. In the following sections the general reaction scheme:

$$\begin{array}{l} \left[{\left({\text{SiO}}_{2}\right)}_{m}\right]-\text{SiO}•+\text{nCO}\to \left[{\left({\text{SiO}}_{2}\right)}_{m}\right]-\text{SiO}•\\ \;+\left[{\left(\text{CO}\right)}_{n}\right]\left(n=1-4\right) \end{array}$$

is analyzed, where the siloxyl radical works as an effective catalyst in CO polymerization. Further analysis will be applied to the release and hydrogenation of the pCO products.

## 2. Computational Methods

The ORCA software (version 4.0.2) (Neese, 2012) was used for all geometry minimizations, potential energy surface (PES) and vibrational frequency analyses using the global hybrid functional PW6B95 (Zhao and Truhlar, 2005) coupled to the split valence triple-ζ def2-TZVPP basis set with two sets of polarization functions (Weigend and Ahlrichs, 2005) and the atom-pairwise dispersion correction energy with Becke-Johnson damping (D3BJ) (Grimme et al., 2010, 2011). The selected level of theory (PW6B95-D3BJ/def2-TZVPP) was shown to be one of the most robust, reliable and accurate theoretical tools in the estimation of general main group thermochemistry, kinetics and non-covalent interactions after the double hybrid functionals (Goerigk et al., 2017). The reliability of the used method is also underlined by the good qualitative agreement between the DFT and MP2-F12 calculations as found in previous works (Fioroni and DeYonker, 2016; Fioroni et al., 2018, 2019).

To compare the DFT data to results when electron correlation effects are considered, the domain-based local pair-natural orbital (DLPNO) approach coupled to the CCSD(T) method [DLPNO-CCSD(T)] (Riplinger and Neese, 2013; Riplinger et al., 2013) in conjunction with the quadruple-ζ correlation consistent basis set cc-pVQZ (Dunning, 1989; Woon and Dunning, 1993) was applied on the first three intermediates representing the first two CO addition reactions (see **Figure 3**). The differences in the electronic energies (ΔE_*el*_) between the two levels of theory are reported in Table 1. The trend between the two PES shows a good agreement, with the DFT results tending to stabilize the intermediates.

**Table 1:** Electronic energies differences (ΔE_*el*_, kcal/mol) at the PW6B95/def2-TZVPP and DLPNO-CCSD(T)/cc-pVQZ level of theory based on the first two CO addition reactions.

**Reaction type**	**Δ${\text{E}}_{el}^{DFT}$**	**Δ${\text{E}}_{el}^{CCSD(T)}$**
(a) 1*st* CO addition, **1**→**2**	−31.0	−26.5
(b) 2*nd* CO addition (TS) **2**→**TS I**	−30.4	−26.3
(c) 2*nd* CO addition **2**→**3**	−40.7	−31.5

To speed up calculations the RI (Resolution of the Identity) (Neese, 2003) and RIJCOSX (Neese et al., 2009) algorithms were used coupling the Coulomb-fitting basis sets def2/J (Weigend, 2006). The NEB (Nudged Elastic Band) method for finding minimum energy paths of transition states was used (Jonsson et al., 1998). To test if the computed structures represent a minimum or a transition state, vibrational frequency calculations within the harmonic approximation were performed on all compound models. The obtained enthalpies (H_*Tot*_=[E_*El*._+E_*ZPE*_+E_*Vib*._+E_*Rot*._+E_*Trans*._]+k_*B*_T) and S values (S_*Tot*_=S_*El*._+S_*Vib*._+S_*Rot*._+S_*Trans*._) were used to estimate the free energies (G) at T = 200 K.

Reaction rates have been estimated by the Eyring relation (T = 200 K):

$$k=\frac{k_{B}T}{h}e^{-\frac{\Delta G}{RT}}$$

Figures were rendered by the program Avogadro (Hanwell et al., 2012).

## 3. Results and Discussion

### 3.1. CO Polymerization

CO polymerization (pCO) is not an “easy” chemical task. Only few examples of CO polymers like oxocarbons (CO)_*n*_ have been obtained by the direct reaction of several CO units (West and Niu, 1970; Seitz and Imming, 1992). Experiments performed at high pressures (GPa) on pure CO, produced a metastable CO polymer (Lipp et al., 2005) structurally characterized by partially interconnected polycarbonyl chains where the monomeric unit of five carbons contains a lactone moiety (Bernard et al., 1998). Further high pressure experiments conducted on Fe(CO)_5_ resulted in a mixture of Fe_2_O_3_ crystals immersed in a polymerized CO (Ryu et al., 2015). Overall, CO polymerization is a difficult reaction if a catalyst is not involved.

**Figure 2:**
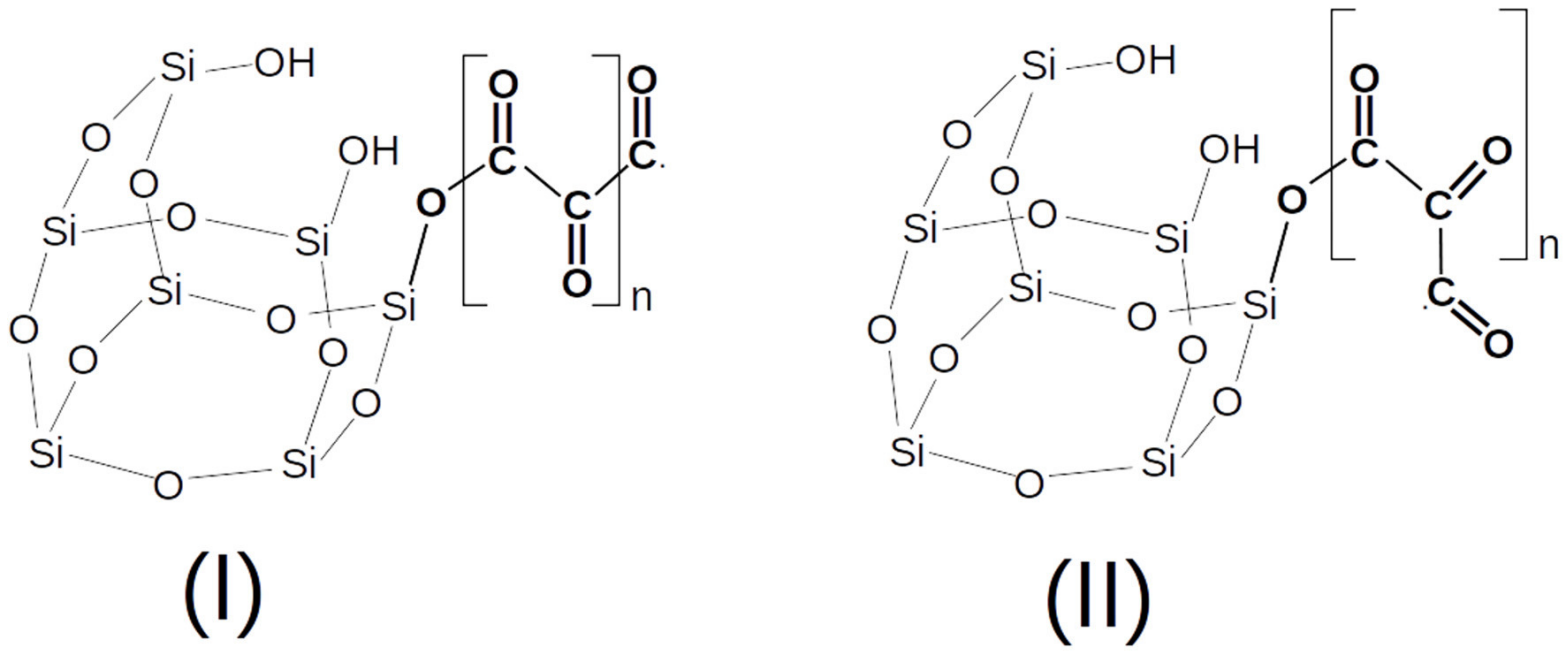
A nascent CO polymer chain (pCO) in the *trans* (I) and *cis* (II) conformers.

**Figure 3:**
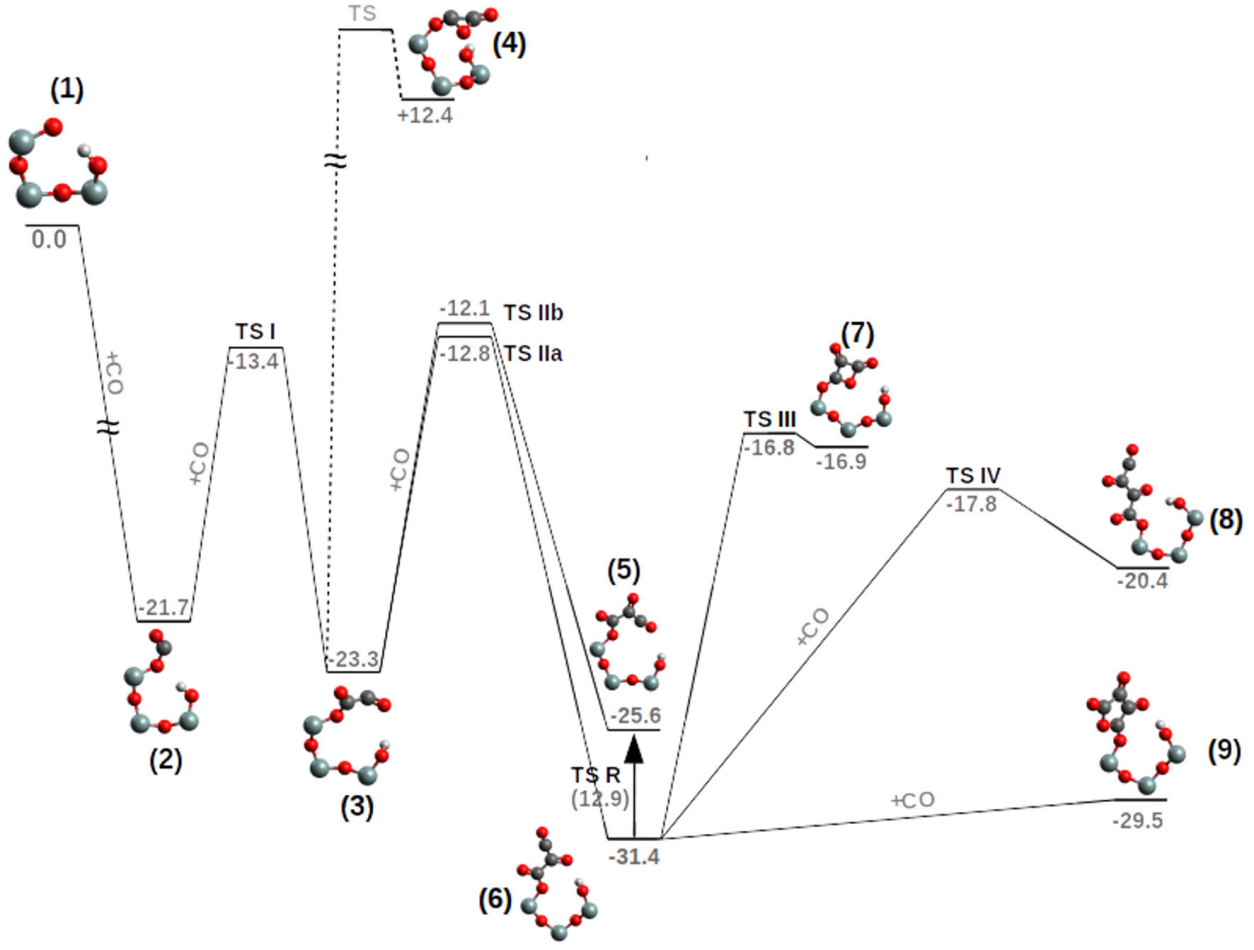
PES (ΔG, kcal/mol, T = 200 K) of the pCO formation on a siloxyl radical center. TS *R*-value (in parenthesis) corresponds to the free energy of activation. For clarity, only half of the POSS moiety is reported and hydrogen atoms on the Si atoms have been deleted.

In Figure 2, a possible example of a nascent CO polymer on a SiO• radical working as a catalytic center is shown with its *trans* and *cis* isomers, while in Figure 3 the calculated PES (T = 200 K) of the following reaction is reported,

$$\begin{array}{l} \left[{\left({\text{SiO}}_{2}\right)}_{m}\right]-\text{SiO}•+\text{nCO}\to \left[{\left({\text{SiO}}_{2}\right)}_{\text{m}}\right]-\text{SiO}-\left[{\left(\text{CO}\right)}_{\text{n}}\right]• \end{array}$$

The selected T = 200 K is a lower bound to the (CO-CO) polymerization to progress efficiently, though the 1*st* addition is barrier-less and the 2*nd* addition can work at lower temperatures (see next paragraphs). Referring to astronomical bodies, we have hypothesized such temperatures can be experienced, for example, by comets where temperature rise periodically by surface heating to release CO and H molecules (Hoang et al., 2019) or by dust particles or greater bodies in the turbulent phase of a proto-planetary disk.

**Figure 4:**
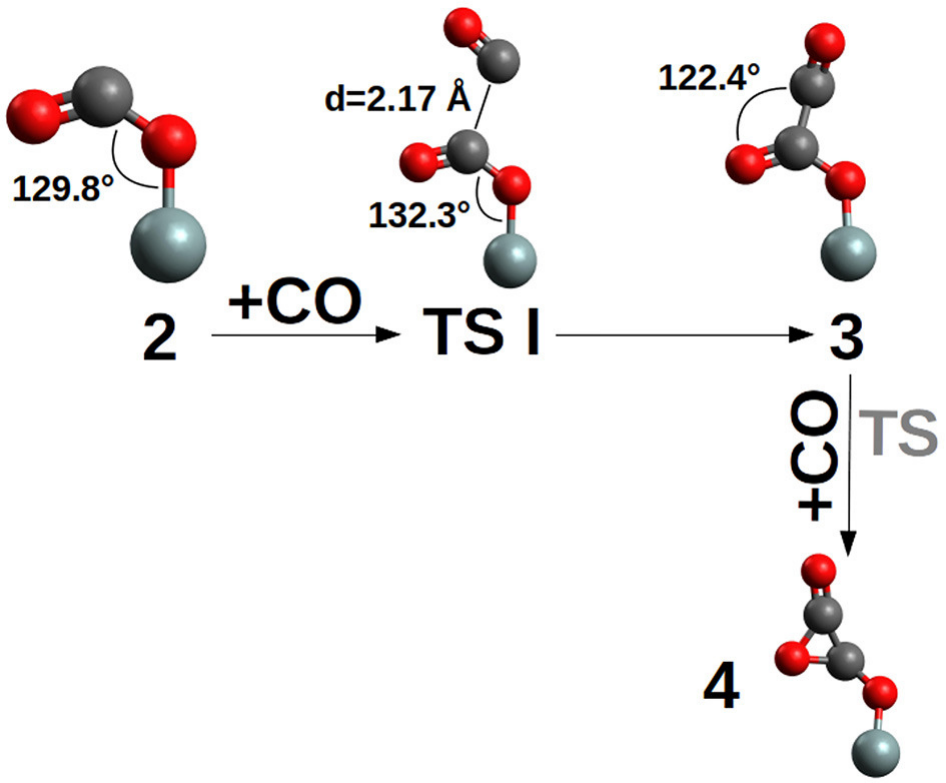
**TS I** and (CO)_2_ products **3** and three-member ring **4**. **TS** was not determined due to the unlikely formation of the three-ring product (see Figure 3). In compound **3** the OCCO dihedral between the two CO is ≈ 96°. For clarity, only the siloxy group of the POSS moiety is shown.

The first CO addition to the siloxyl center, compound **1**, to give the siloxy-carbonyl compound **2**, is thermodynamically favored by ΔG = −21.7 kcal/mol and no free energy of activation is observed (see Figure 4 and Supplementary Figure 1).

**Figure 5:**
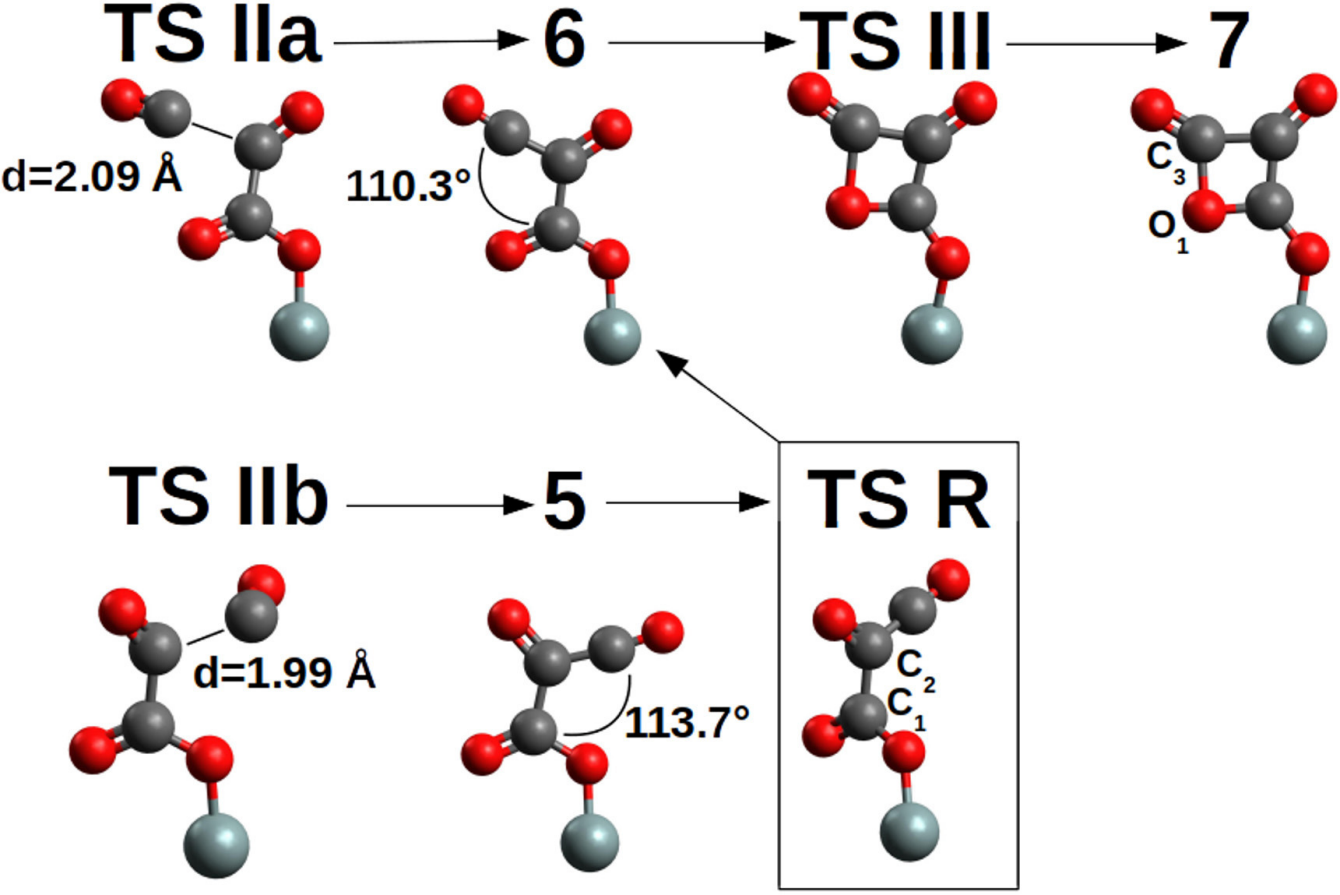
**TS II** and (CO)_3_ products **5**-*cis* and **6**-*trans* together with the four-member ring **7** of the pCO. **TS R** connects **5**-*cis* and **6**-*trans* by a rotation of the C_1_-C_2_ axis. **TS III** is a late TS characterized by a bond length C_3_-O_1_=1.63 Å, while in product **7**, 1.49 Å. For clarity, only the siloxy group of the POSS moiety is shown.

The second CO addition, **2**→**3**, must overcome an activation free energy of ΔG^‡^=+8.3 kcal/mol (k=3.54*10^3^ s^−1^M^−1^) (**TS I**), while the thermodynamics is, relative to compound **2**, slightly favored by ΔG = −1.6 kcal/mol. Furthermore, compound **3** is characterized by a -Si-O-CO-CO• skeleton which has the potential to perform a three-ring closure reaction to end up in an enthalpically “ring strained” oxalic anhydride precursor (**4**) which is, not surprisingly, thermodynamically strongly disfavored by ΔG = +35.7 kcal/mol. The third CO addition to compound **3**, Figure 5, can follow two different reaction channels by two different transition states, **TS IIa** and **TS IIb**. In fact, compound **3** is characterized by an OCCO dihedral of ≈96° (see Figure 4) and the CO attack can be performed via the two opposite sides to obtain the *cis-trans* products (Figure 5). The lowest in energy, **TS IIa**, with a ΔG^‡^=+11.2 kcal/mol (k = 2.40 s^−1^M^−1^) forms compound **6**, while **TS IIb** with a ΔG^‡^=+10.5 kcal/mol (k = 1.40 * 10^1^ s^−1^M^−1^) ends up in compound **5**.

Product **6** is the pCO *trans* form, which is thermodynamically favored over the **5**-*cis* form by ΔG = −5.8 kcal/mol. As a consequence the **5**-*cis* is both thermodynamically as well as kinetically disfavored compared to the **6**-*trans*. Interconversion between the **5**-*cis* and **6**-*trans* is possible by performing a 180° rotation of the OC_1_C_2_O dihedral (Figure 5) determining a **TS R**, ΔG^‡^ = +12.9 kcal/mol (k = 3.34 * 10^−2^ s^−1^M^−1^). This interconversion further cements product **6** as the expected product. Furthermore by ring closure, the precursor of the di-cheto form of oxetane (compound **7**) is formed, characterized by a very late TS (**TS III**) and thermodynamically uphill by ΔG = +14.5 kcal/mol.

**Figure 6:**
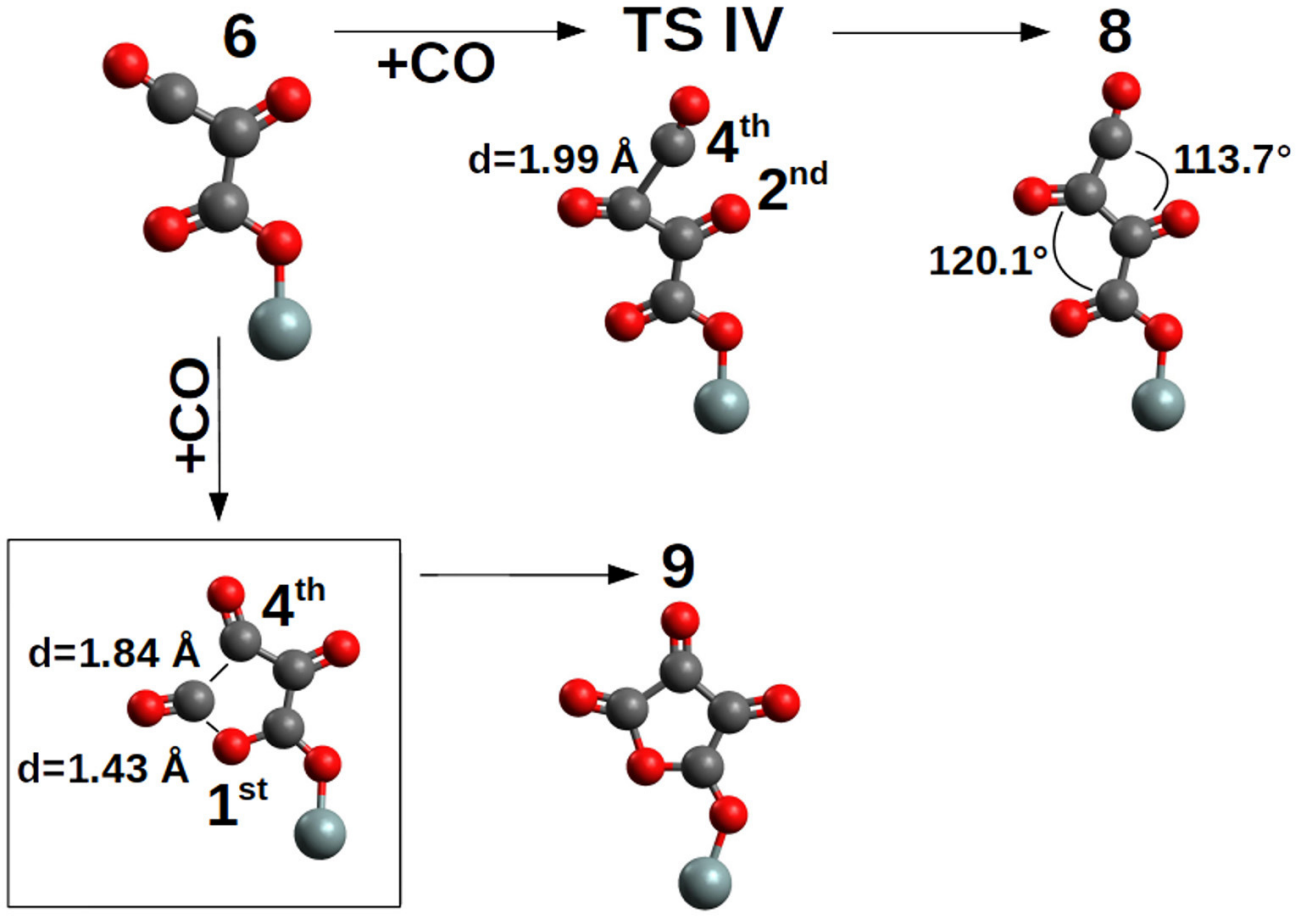
Elongation and cyclization of the pCO chain by addition of the 4*th* CO. For clarity, only the siloxy group of the POSS moiety is shown.

The addition of the fourth CO on the **6**-*trans* proceeds by two possible orientations (Figure 6). First, with the C→O axis parallel to the second C→O axis of the nascent pCO (Figure 6) and by **TS IV** (ΔG^‡^ = +14.6 kcal/mol, k = 4.63 * 10^−4^ s^−1^M^−1^) compound **8**-*trans* (ΔG = +11.0 kcal/mol) is formed, or with the C→O axis parallel to the first C→O axis of the nascent pCO (Figure 6) and by cyclization and no TS, **9** is formed. The path between compounds **6** and **9** is characterized by a barrierless concerted step where the C-C and C-O bonds are formed (see Supplementary Figure 2).

Compound **9** is an epoxo five member ring precursor of 1,4-epoxy-1,2,3-butanone, seemingly a natural consequence of the pCO chain with four CO, although slightly disfavored by ΔG=+1.9 kcal/mol relative to **6**-*trans*, which is the most stable pCO form bond to the siloxyl center. However, at the considered T = 200 K, population densities of compound **9** will be enough to further proceed in different reaction channels such as CO addition or hydrogenation (see next section). Furthermore, in Figure 3, the elongation of the pCO chain from the four CO units seems to be thermodynamically disfavored with compound **8**-*trans* ΔG=+11.0 kcal/mol above compound **6**-*trans*.

In most of the reaction steps, a hydrogen bond between one of the SiOH groups and the nascent CO polymer, is present (see Figure 3). The distance varies from ≈1.85 Å (strong H-bond) as in the free siloxyl radical **1**, the important intermediates **6** and the final products **8** and **9**, while in the intermediates **2**, **3**, and **5** the H-bond is weak or not existing (2.20–2.70 Å). The H-bond importance resides in its ability to better stabilize compound **6** over **5** and to reduce the rotational entropy associated to the nascent CO polymer.

Potentially the cyclic compound **9** can accept one CO on the C bond to the siloxy group due to its radical character, opening the possibility to “branch” the pCO. However, the reaction is thermodynamically strongly disfavored by ΔG=+30.0 kcal/mol (see Supplementary Figure 3). Notably, product **9** has the same structure as the pCO obtained by CO high pressure experiments, where the 5-ring lactone moiety is connected by CO units (Bernard et al., 1998; Lipp et al., 2005).

### 3.2. pCO Release and Hydrogenation

Once the pCO chains or rings are formed, their hydrogenation and/or release from the surface are important processes to be analyzed. Hydrogen has three important characteristics to be considered: **(a)** it is the most abundant element in the universe and can, with high probability, react with the pCO; **(b)** H addition is characterized by very low or no activation energy; **(c)** the thermodynamics is highly exothermic i.e., favored.

Furthermore, while the neutral oxocarbons are unstable species (Schröder et al., 1999; Jiao et al., 2001; Corkran and Ball, 2004; Lunny et al., 2018) from the addition of H atoms or electron “injection,” stable compounds are obtained (Seitz and Imming, 1992) such as the cyclic “deltic acid” (C_3_O_3_H_2_, 2,3-dihydroxycycloprop-2-ene-l-one) and its di-anion (C_3_O_3_)^2−^, “squaric acid” (C_4_O_4_H_2_, 3,4-dihydroxycyclobut-3-en- 1,2-dione) and the correlated di-anion (C_4_O_4_)^2−^, C_5_O_5_H_2_ “croconic acid” (4,5-Dihydroxy-4-cyclopentene-l,2,3-trione) and C_6_O_6_H_2_ “rhodizonic acid” (5,6-dihydroxycyclohex-5-ene-1,2,3,4-tetrone). As such, the pCO hydrogenation is an interesting chemistry due to the stabilization effects induced by the H addition and will be a subject of future study.

Unfortunately, the complexity of the pCO PES hydrogenation scales up fast. For example, by considering the smallest [(SiO_2_)_*m*_]-SiO-[(CO)_2_]• model, its full hydrogenation would give an interesting product like ethylene glycol: [(SiO_2_)_*m*_]-SiO-[(CO)_2_]•+6H→[(SiO_2_)_*m*_]-Si-O•+(CH_2_OH)_2_. A detailed PES analysis would be computationally quite demanding. However, selected examples can be analyzed to extract key answers on the general behavior and main characteristics of the pCO hydrogenation. As general rule, the pCO hydrogenation follows two main classes of reactions:

**I)** a radical-radical reaction, where the H atom interacts with a radical species, for example [(SiO_2_)_*m*_]-SiO-[(CO)_*n*_]•;

**II)** a radical-closed shell reaction, where the H atom reacts with closed shell species, for example, [(SiO_2_)_*m*_]-SiO-[(CO)_*n*−1_CHO]. The radical-radical and radical-closed shell reactions will be alternating to reach the final fully, or partially, hydrogenated products.

Radical-radical reactions generally have very low or no activation energies. The validity of such assertion was analyzed by considering the first hydrogenation step (see Supplementary Material) on some representative compounds like: [(SiO_2_)_*m*_]-SiO-[CO]•+H→[(SiO_2_)_*m*_]-SiO-[CHO]; [(SiO_2_)_*m*_]-SiO-[(CO)_2_]•+H→[(SiO_2_)_*m*_]-SiO-[(CO)-CHO]; (cyclic) [(SiO_2_)_*m*_]-SiO-[(CO)_4_]•+H→ (cyclic) [(SiO_2_)_*m*_]-SiO-[CHO-(CO)_3_]. All reactions are, not surprisingly, barrier-less. Interestingly, due to the presence of the aldehyde group, the surface will be “activated” toward nucleophiles or radicals.

Another group of interesting radical-radical reactions are the hydrogen addition to the pCO carbons and oxygens or to the oxygen of the siloxy group. In particular, the [(SiO_2_)_*m*_]-SiO-[(CO)_2_)]• moiety was analyzed in detail to illustrate the possible reaction paths triggered by H addition. As a first case study, the hydrogenation of the siloxy oxygen with a ΔG = −42.7 kcal/mol allows the release of the unstable ethylen-dione (Table 2, reaction a). However, another four hydrogenation sites characterized by the two oxygens and the two carbons of the (CO)_2_ molecule are possible (Table 2, reactions b, c, d, e). All reactions are thermodynamically downhill with the hydrogenation of the carbons preferred to the oxygens due to their higher radical character (see Supplementary Figure 4).

**Table 2:** ΔG reaction energies (kcal/mol, T = 200 K).

**Reaction type**	**Energy**
**Hydrogenation reactions of the oxygens**	
(a) Si**O**-[(CO-CO)]•+H→ -Si**O**H + (CO)_2_^*a*^	−42.7
(b) SiO-[(CO-C**O**)]•+H→ -[SiO-(CO-C**O**H)]	−32.2
(c) SiO-[(C**O**-CO)]•+H→ -[SiO-(C**O**H-CO)]	−50.5
**Hydrogenation reactions of the carbons**	
(d) SiO-[(CO-**C**O)]•+H→ -[SiO-(CO-**C**HO)]	−84.7
(e) SiO-[(**C**O-CO)]•+H→ -[SiO-**C**HO] + CO	−85.8
(f) SiO-[(**C**O-(CO)_2_)]•+H→ -[SiO-**C**HO] + (CO)_2_^*a*^	−32.9
(g) SiO-[(**C**O-(CO)_3_)]• (linear) +H→ -[SiO-**C**HO] + (CO)_3_^*b*^	−28.6
**Release of the oxocarbons**	
(h) SiO-[(CO)_2_]•→ -Si-O• + (CO)_2_^*a*^	+72.5
(i) SiO-[(CO)_3_]•→ -Si-O• + (CO)_3_^*b*^	+96.0
(j) SiO-[(CO)_4_]•→ -Si-O• + (CO)_4_ (linear)^*c*^	+96.1
**Hydrogenation reactions of the last carbon**	
(k) SiO-[(**C**O]•+H→ -[SiO-**C**HO)]	−91.8
(l) SiO-[(CO-**C**O)]•+H→ -[SiO-(CO-**C**HO)]	−84.7
(m) SiO-[(CO)_2_-**C**O]•+H→ -[SiO-(CO)_2_-**C**HO)]	−65.7
(n) SiO-[(CO)_3_-**C**O]• (linear) +H→ -[SiO-(CO)_3_-**C**HO)]	−67.4

a *Triplet state, singlet unstable in agreement with Lunny et al. (2018)*.

b *Singlet state, ΔG_S = 3_-ΔG_S = 1_=+7.8 kcal/mol*.

c *Triplet state, ΔG_S = 3_-ΔG_S = 1_=-5.4 kcal/mol. Bold labeled atoms=reaction site*.

The hydrogenation of the first carbon bond to the siloxy unit will interestingly always result in the release of the following (CO)_*n*−1_ moiety (Table 2, reactions e, f, g) leaving a very reactive aldehyde group on the surface. These results suggest that hydrogenation of the first carbon releasing the free oxocarbon and an aldehyde group on the surface is the most favored one. The hydrogenation of the carbon of the second CO (reaction d, Table 2) gives the radical precursor of glyoxal (CHO-CHO), which release is thermodynamically strongly disfavored by ΔG=+93.3 kcal/mol. The importance of glyoxal, as a potential product and being the smallest di-aldehyde, lies in its ability to react with urea resulting in five- and six-member non-aromatic heterocycles able to self-assemble in chains or in dendritic-like structures with masses up to 1 kDa (Lavado et al., 2019).

After the first hydrogenation, the next hydrogen will react with a neutral-closed shell molecule. As a case study, two different PESs based on the [(SiO_2_)_*m*_]-SiO-[(CO)-CHO] model were analyzed:

**(a)** the hydrogen attacks the siloxyl oxygen, to release the glyoxal radical as final product: [(SiO_2_)_*m*_]-SiO-[(CO)-CHO]+H→[(SiO_2_)_*m*_]-SiOH+•CO-CO. The reaction is thermodynamically disfavored, ΔG = +18.2 kcal/mol, and the TS shows a ΔG^‡^ = +25.4 kcal/mol (k=7.32 * 10^−16^ s^−1^M^−1^) high in energy, a quite slow reaction even at room temperature.

**Figure 7:**
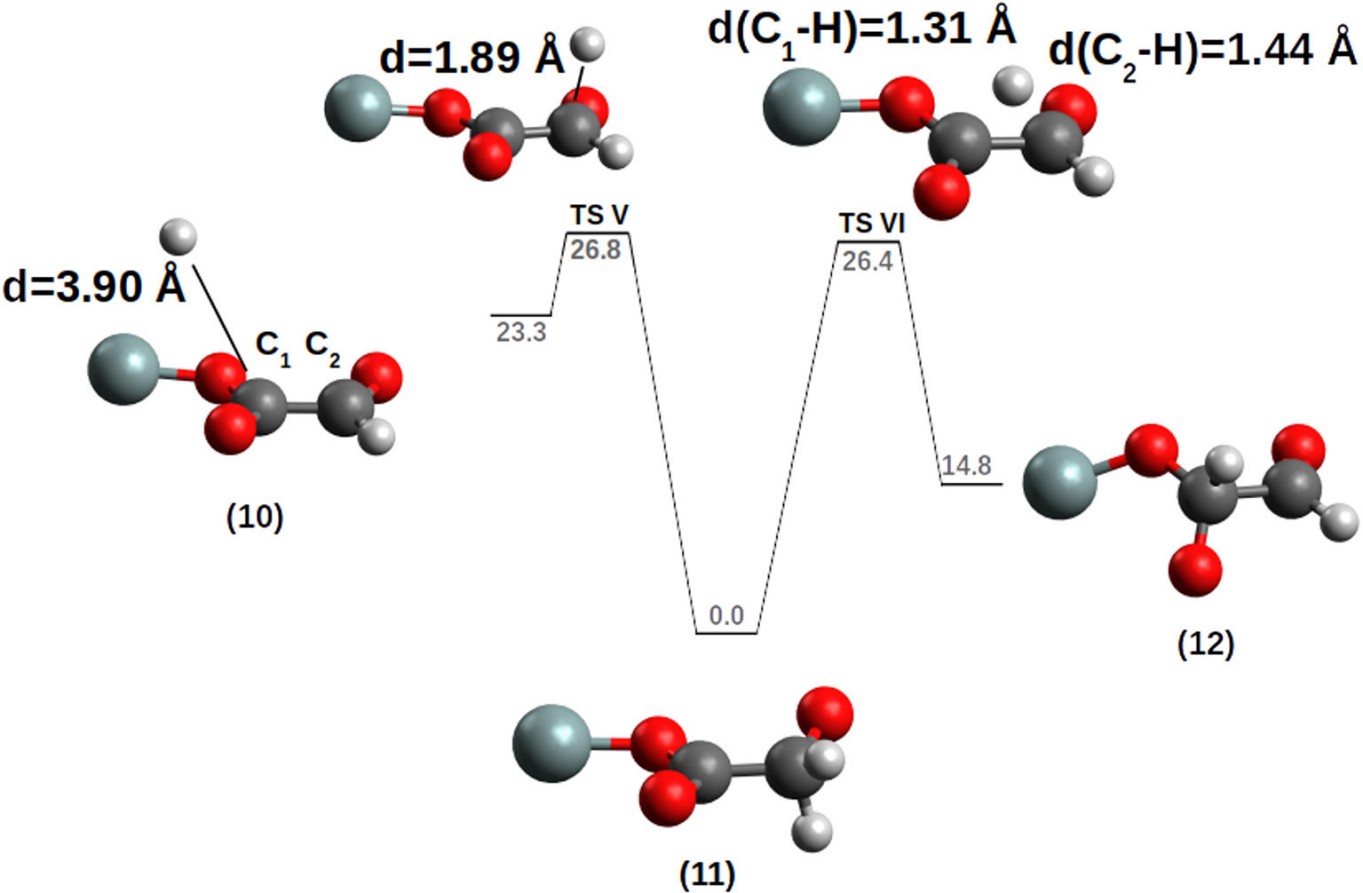
Hydrogenation of the [CO-(CHO)] chain. The PES was calculated by moving the H atom toward the C_1_ carbon. For clarity, only the siloxy group of the POSS moiety is shown.

**(b)** the second hydrogen addition was conducted on the carbon atom bound to the siloxyl oxygen, expecting a reaction like: [(SiO_2_)_*m*_]-SiO-[(CO)-CHO]+H → [(SiO_2_)_*m*_]-SiO•+CHO-CHO. Most interestingly, the reaction PES forms a thermodynamically and kinetically preferred radical alkoxide instead of the di-aldehyde (glyoxal) (see Figure 7). Starting from compound **10** the incoming hydrogen prefers to attack the external carbon C_2_ to give the radical alkoxide **11** passing through **TS V** with a ΔG^‡^=+3.5 kcal/mol (k = 6.24 * 10^8^ s^−1^M^−1^). To reach the SiO-CHO-CHO dialdehyde (compound **12**), the reaction **11** → **12** is thermodynamically disfavored, ΔG = +14.8 kcal/mol, with an activation barrier of ΔG^‡^ = +26.4 kcal/mol (k = 5.91 * 10^−17^ s^−1^M^−1^) (**TS VI**). **TS VI** corresponds to an internal H transfer from the CH_2_ group to the C of the SiO-CO moiety.

The following hydrogenation reactions will alternate a radical-radical to a radical-closed shell mechanism, releasing the fully hydrogenated products (alcohols) or, less likely, partially hydrogenated compounds like aldehydes. In principle, considering the [(CO)_*n*_] linear compounds with *n* = 2,3,4, the possible final products (differently populated depending from their parental (CO)_*n*_ thermodynamic stability) are ethan-1,2-diol (ethylen glycol), propane-1,2,3-triol (glycerol) and butane-1,2,3,4-tetraol, the last in its four diastereomeric forms including erythritol and threitol. Note that by the full hydrogenation of the cyclic 1,4-epoxy-1,2,3-butanone (**9**) a furanose ring is obtained. For n=1 methanol is produced, as already analyzed by Goumans et al. (2008).

Further interesting reactions are produced by the direct release of the pCO chains into the gas phase.

$$\begin{array}{l} \left[{\left({\text{SiO}}_{2}\right)}_{\text{m}}\right]-\text{SiO}-\left[{\left(CO\right)}_{n}\right]•\to \left[{\left({\text{SiO}}_{2}\right)}_{m}\right]-\text{Si}-\text{O}•+{\left(\text{CO}\right)}_{\text{n}} \end{array}$$

Not surprisingly free energy values are very high (see Table 2, reaction h, i, j) due to the strong bond connecting the siloxy unit to the pCO carbon (SiO-C), characterized by an O-C bond energy of ≈+85.0 kcal/mol (Rumble, 2020). Such unfavorable thermodynamics will inhibit the release of the free pCO due to the relatively weak bond energies between the CO units (see Figure 3), in agreement with previous theoretical and experimental analysis of (CO)_2_ oxocarbon (ethylen dione) and oxocarbons up to *n* = 6 (Schröder et al., 1999; Jiao et al., 2001; Corkran and Ball, 2004; Lunny et al., 2018).

By checking Table 2, the reactivity of the pCO chains shows some interesting characteristics. In the hydrogenation of the terminal CO carbon (see Table 2, reaction k, l, m, n), ΔG is inversely proportional to the pCO chain length, to reach a “plateau” in longer chains. Such behavior can be correlated to the spin density on the last carbon, decreasing with the increasing length of the chain (see Supplementary Material). From these results, hydrogenation reactions on longer chains may be effectively performed on the oxygen atoms.

Another interesting reaction is the hydrogenation of the first CO carbon leaving a SiO-CHO on the surface and releasing a (CO)_*n*_ into the gas phase (*n*>1) (see Table 2, reaction e, f, g). The ΔG is always negative though for *n* = 3-4 the values are lower compared to *n* = 2. Again such results can be correlated in the decrease of the spin density on the first CO carbon when part of longer chains. However, in the (CO)_2_ case, the hydrogenation of the first CO carbon, releasing a CO molecule, is slightly preferred to the hydrogenation of the second CO carbon (see Table 2, reaction d, e). Though the spin density would suggest the second carbon to be more reactive, the balance between the enthalpic (mainly bond formation) and the entropic terms (mainly correlated to the release of one CO) favors the release of the CO by the hydrogenation of the first CO. The difference in entropy between the two reactions is ΔΔS_*e*−*d*_=+1.2 kcal/mol (CO release) while the enthalpy remains practically constant ΔΔH_*e*−*d*_=0.04 kcal/mol (balanced between the strong H-C bond formation and the low energy C-C disruption).

In case of longer chains the CO_*n*_ release is still entropically favored, though with a contribution of only a few kcal/mol associated to the roto-translational entropy of the (CO)_*n*_ chains. The enthalpic factor will determine the reaction profile proportional to the pCO spin density.

Another set of reactions, same as discussed for the (CO)_2_ molecule, is the release and hydrogenation of the five member ring precursor of the 1,4-epoxy-1,2,3-butanone (Table 3). The release of the newly synthesized five-member ring bond to the siloxy group has a huge free energy penalty of ΔG=+115.9 kcal/mol (Table 3, reaction a), underlining the near double bond character of the SiO-C bond.

**Table 3:** ΔG reaction energies (kcal/mol, T=200 K).

**Reaction type**	**Energy**
(a) Si-[**O-C**_*ring*_]•→ -Si-**O**• + **:C**_*ring*_	+115.9
(b) Si-[**O**-C_*ring*_]•+H→ Si**O**H + (:C_*ring*_)	+59.8
(c) Si-[O-**C**_*ring*_]•+H→ [SiO-**C**_*ring*_H]	−59.1
(d) Si-[O-**C**_*ring*_H]+H→ -Si-O• + H_2_**C**_***ring***_	−1.8
(e) Si-[**O-C**_*ring*_H]→ -Si-**O**• + •**C**_*ring*_H	+73.9
(f) Si-[**O**-C_*ring*_H]+H→ Si-**O**H + •C_*ring*_H	−41.3

*The bold labeled atoms are the reaction sites. C_ring_ = first carbon bond to the siloxy group precursor of 1,4-epoxybutane-1,2,3-dione (H_2_C_ring_); •C_ring_H = radical, :C_ring_ = carbene*.

Focusing on the five-member ring hydrogenation reactions (Table 3, reaction b–f), the hydrogenation of the siloxy oxygen would release (reaction b) the carbene of the five-member ring if not disfavored by ΔG=+59.8 kcal/mol, while the hydrogenation of the carbon (reaction c) has a ΔG = −59.1 kcal/mol. A further hydrogenation on the CH of the five-member ring will release the final product 1,4-epoxy-1,2,3-butanone, thermodynamically favored by ΔG = −1.8 kcal/mol. The direct dissociation of the SiO-CH(ring) (reaction e) releasing the two radicals of the 1,4-epoxy-1,2,3-butanone and the POSS-siloxyl is thermodynamically unfavorable by ΔG=+73.9 kcal/mol, a free energy value that underlines the single bond character of the SiO-CH. Finally, the hydrogenation of the siloxy oxygen (reaction f) would release the 1,4-epoxy-1,2,3-butanone radical, thermodynamically favored by ΔG = −41.3 kcal/mol. Summarizing, the release of the 1,4-epoxy-1,2,3-butanone radical is preferred to the fully hydrogenated form, due to the hydrogenation preference toward the oxygen rather than the carbon of the SiO-CH unit. Once released, the 1,4-epoxy-1,2,3-butanone radical will be a highly reactive species able to be fully hydrogenated or to react promptly with other organic substrates.

## 4. Conclusions

The aim of the study is to understand how a silica surface decorated with siloxy radicals chemically behaves in presence of CO molecules, as CO is one of the most abundant carbon carriers in the Universe. In fact, while the H_2_O case is well analyzed having important consequences in the chemical synthesis and electronics industries, the CO case is less studied. By means of quantum chemistry (DFT; PW6B95/def2-TZVPP) the general reaction:

$$\begin{array}{l} \left[{\left({\text{SiO}}_{2}\right)}_{\text{m}}\right]-\text{SiO}•+\text{nCO}\to \left[{\left({\text{SiO}}_{2}\right)}_{\text{m}}\right]-\text{SiO}•+{\left(\text{CO}\right)}_{\text{n}}\left(\text{n}=1-4\right) \end{array}$$

at T=200 K is analyzed, where the (CO)_*n*_ is the CO polymerization product (pCO) and the [SiO_*m*_]-SiO• moiety works as an effective catalyst by the SiO• (siloxyl radical) group.

The proposed catalytic model is based on three assumptions. First, that siliceous rocks in both crystalline or amorphous states show defective centers such as siloxyl radicals. This hypothesis has its foundations in industrial catalysis or geochemistry, where defective centers are known to play a fundamental role in the catalysts activity. Second, that the amorphous siliceous surface is appropriately modeled by a silica-POSS (polyhedral-silsesquioxane) moiety. Because the SiO_4_ or [(SiO_2_]_*x*_ units and skeleton are present in a siliceous lattice, the chemistry of a pure silica model will be reasonably transferable to a silicate when only the SiO• chemistry is considered. Third, that the second phase is represented by CO molecules, an abundant C_1_ building block in space.

Furthermore, the T = 200 K was selected being a lower bound to the (CO-CO) polymerization to progress efficiently, though the 1*st* addition is barrier-less and the 2*nd* addition can proceed at lower temperatures (see next paragraphs). It has been hypothesized that such temperatures can be experienced, for example, by comets where temperature rise periodically by surface heating to release CO and H or by dust particles or greater bodies in the turbulent phase of a proto-planetary disk.

Our findings have multiple consequences in astrochemistry:

**(I)** due to the barrier-less addition of the first CO to the SiO• center and the strong thermodynamic driving force (−21.7 kcal/mol), the SiO-CO• group can be formed at very low T, saturating the siloxyl centers present on siliceous minerals. Furthermore, the SiO-CO• bond stability will shift the CO release from the surface at higher T compared to a neat CO ice;

**(II)** the sequential addition of four CO catalyzed by the siloxyl radical produces a set of pCO composed by two, three and four CO monomers (C-C bond based), and the last precursor is 1,4-epoxy-1,2,3-butanone characterized by a lactone moiety;

**(III)** the pCO linear chains (CO)_2_ and (CO)_3_ have a bond strength of a few kcal/mol though kinetically stabilized by activation free energies of ≈ ΔG^‡^ ≤ +19.0 kcal/mol. The SiO-(CO)_4_ ring (precursor of 1,4-epoxy-1,2,3-butanone) is formed by a barrier-less cyclization reaction, but is thermodynamically slightly disfavored by ΔG = +1.9 kcal/mol compared to the linear precursor SiO-(CO)_3_, which is the most stable pCO form bound to the siloxy center. However, due to the low energy difference, the population of the SiO-(CO)_4_-ring should be enough (T = 200 K) to participate in subsequent reactions;

**(IV)** the release of the pCOs, i.e., breaking the SiO-C bond, has a not surprisingly high free energy penalty, +75.0 ≤ ΔG ≤ +115.0 kcal/mol. Such high free energies will inhibit the presence of free pCO due to the intrinsic instability of the oxocarbons (CO)_*n*_ as experimentally known for the *n* = 2–6 cases because of the relative weak bond energies between the CO units [see point **(III)**];

**(V)** considering hydrogenation reactions, the analysis was focused on the [SiO_*m*_]-SiO-(CO)_2_ and [SiO_*m*_]-SiO-(CO)_4_ systems. Hydrogen has three important characteristics under our consideration: first, it is the most abundant element in the universe. Second, H addition is characterized by very low or negligible kinetic barriers, and third, the thermodynamics is favorable and highly exothermic.

In the case of (CO)_2_, the findings are:

**(Va)** the hydrogenation of the siloxy oxygen determines the release of the unstable (CO)_2_ (ethylen-dione) favored by ΔG = −42.7 kcal/mol;

**(Vb)** referring to the selective hydrogenation of the (CO)_2_ carbons or oxygens, carbons are energetically preferred due to their radical character. Noticeably, the hydrogenation of the first carbon bond to the siloxy group (SiO-**C**) results in the release of the following (CO)_*n*−1_ chain leaving an extremely reactive aldehyde group, SiO-(CHO), on the surface. This result has important consequences because other chemical species can react with the aldehyde group fixed on the surface.

Regarding the (CO)_4_ ring the findings are:

**(Vc)** the hydrogenation of the siloxy oxygen would release the ring if not for a free energy penalty of ΔG=+59.8 kcal/mol, while the hydrogenation of the carbon bond to the siloxy group (SiO-**C**) is favored by ΔG=-59.1 kcal/mol.

After carbon hydrogenation, the splitting of the Si**O**-**C**H bond is unfavorable with a ΔG=+73.9 kcal/mol, while the hydrogenation of the siloxy oxygen will release the ring 1,4-epoxy-1,2,3-butanone radical [•H(C_4_O_4_)], favored by ΔG = −41.3 kcal/mol.

The general findings suggest the release of 1,4-epoxy-1,2,3-butanone radical from the surface as the most favorable reaction path. It should be noticed that the product 1,4-epoxy-1,2,3-butanone has the same structure as the pCO obtained by CO high pressure experiments.

**(VI)** in principle, by the full hydrogenation of the considered oxocarbons, final products such as methanol, ethan-1,2-diol (ethylen glycol), propane-1,2,3-triol (glycerol) and butane-1,2,3,4-tetraol, the last in its four diastereomeric forms including erythritol and threitol, are obtained. By the full hydrogenation of the cyclic 1,4-epoxy-1,2,3-butanone a furanose ring is obtained. Possible synthetic routes of the aforementioned alcohols have been obtained by the analysis of the consecutive hydrogenation reactions: [SiO_*m*_]-SiO-(CO)_2_]+H→[SiO_*m*_]-SiO-CO-CHO+H→[SiO_*m*_]-SiO-CO-CH_2_O. Results show radical alcoxides are preferred intermediates paving the way toward polyols synthesis. As general rule, the hydrogenation reactions alternates between a radical-radical to a radical-closed shell mechanism.

**(VII)** pCOs IR signatures are important for an experimental confirmation and characterization. Unscaled harmonic vibrational spectra of the POSS-(CO)n polymers are reported using DFT (see Supplementary Material). Si**O-C** stretching frequencies lie between 7.31 and 8.47 μm (blue shift in longer chains), while terminal COs are characterized by a CO stretch between 4.57 and 5.01 μm (blue shift in longer chains). Internal C stretching frequencies are identified by an IR signature comprised between 5.41 and 6.58 μm, dependent on the *cis-trans* polymer conformations. Cyclic compounds show different shifts, with the Si**O-C** stretching frequencies within the 7.31–7.64 μm interval, while the COs inside the rings have a CO stretch between 5.05 and 6.35 μm.

**(VIII)** the oxocarbon-polyketones-lactone 1,4-epoxy-1,2,3-butanone radical can be an important building block in further polymerization reactions and/or open ring reactions with H (aldehydes, polyols) or CN (chetonitriles), resulting in highly reactive multi-functional compounds contributing to COM synthesis.

“Easy” pCO synthesis is predicated on the reactive siloxyl radical being able to chemically activate a relatively inert CO molecule. Furthermore, CO is only one of the possible interesting substrates as, for example, H_2_O or CN, are able to react with the radical SiO-[(CO)_*n*_]• resulting in a complex surface chemistry with implications to be further analyzed by our group.

## Data Availability Statement

The raw data supporting the conclusions of this article will be made available by the authors, without undue reservation.

## Author Contributions

MF conceived the presented idea and performed the numerical simulations. ND verified the computational methods/results and supervised the findings of this work. Both authors discussed the results, methods, and conclusions, and contributed to the final manuscript.

## Conflict of Interest

The authors declare that the research was conducted in the absence of any commercial or financial relationships that could be construed as a potential conflict of interest.
