# Supplementary material for: Complex Organic Matter Synthesis on Siloxyl Radicals in the Presence of CO
Source: Front Chem. 2021 Feb 1;8:621898. doi: 10.3389/fchem.2020.621898 (PMC7882687; doi:10.3389/fchem.2020.621898)
Supplement: Supplementary file 1 [file Image_1.pdf]

Supplementary Material

Complex Organic Matter Synthesis on Siloxyl Radicals in the Presence of CO

Marco Fioroni, Nathan J. DeYonker

Department of Chemistry, 213 Smith Chemistry Building, The University of Memphis, Memphis, TN, USA, 38152

E-mail: mfioroni@memphis.edu; ndyonker@memphis.edu

Supplementary Material

Index

1) ORCA Input Template

2) XYZ

3) Figures S1, S2, S3, S4

4) IR data

5) Spin densities data

6) PES Hydrogenation

7) Figure 7 XYZ

Supplementary Material

ORCA Input Template

```
! UKS RIJCOSX PW6B95 def2-TZVPP def2/J SmallPrint TightSCF MORead

! TightOPT

! UNO

! UCO

! GRID6

! GRIDX6

! D3BJ

! KDIIS

%scf Maxiter=350

    end

%moinp "BP.gbw"

%geom inhess Read

     InHessName "FREQ.hess"

end

%base "OPT"

* xyzfile 0 2 BP.xyz


$new_job

%MaxCore 2000

! UKS RIJCOSX PW6B95 def2-TZVPP def2/J SmallPrint TightSCF MOREad

! UNO

! UCO

! GRID6

! GRIDX6
```

Supplementary Material

```
! D3BJ

! NumFreq

! KDIIS

%moinp "OPT.gbw"

%scf Maxiter=150

     end

%base "FREQ"

%freq Temp 10, 30, 100, 200, 298.15

      CentralDiff true

      Increment 0.0030

      end

* xyzfile 0 2 OPT.xyz
```

Supplementary Material

XYZ

CO

M=1

O 0.97455974383834 1.38160154499780 -0.04235993362382

C 0.36921111023869 0.43867869354435 -0.01506594483112

Glyoxal

M=1

C -0.27424824910262 0.05792650610292 -0.01428148275180

C 1.23718312742125 0.16312822168896 0.01428424822819

O 1.78504703415068 1.19992197238628 0.24615878672256

O -0.82212122757923 -0.97888686004505 -0.24616110664528

H 1.77214970399696 -0.77659086222000 -0.19192164209231

H -0.80921978169626 0.99763403992856 0.19192133137292

Glyoxal radical

M=2

C -0.31645557966963 0.16040755495610 0.00727374870550

C 1.00572556493102 0.55395288572973 0.09993841410468

O 2.11446452435803 0.83703792297322 0.16728005800524

O -0.58805294964658 -1.02543434867423 -0.25511786924126

H -1.04840155997285 0.95092598501518 0.18058564842583

Supplementary Material

$(CO)_2$ Linear

M=3

C	-3.52743475173252	-1.99459583761923	-0.71338857613638
C	-3.51979538594743	-1.90862748350780	-1.98821627246416
O	-3.53447838434197	-2.07388321692450	0.46315611308663
O	-3.51275147797808	-1.82934346194847	-3.16476126448608

$(CO)_3$ Linear

M=1

C	-1.73846227767726	0.62126285703106	0.37859400939621
C	-0.49555532817842	1.05225618010767	-0.05396969770789
O	-2.74237926432451	0.15096647675746	0.65091512011770
O	0.25709191528355	0.39758699223170	-0.83850066291843
C	-0.15903265534688	2.28054441619847	0.49382863358513
O	0.25461944339687	3.27859065378115	0.86215238548508

Supplementary Material

$(CO)_3$ Linear

M=3

C	-1.71045473513489	0.74436641474916	0.43077440036442
C	-0.42341816851701	0.99112209755917	-0.12268692581928
O	-2.70655004576392	0.20931055341724	0.66710298045403
O	0.29833052682778	0.35155956788786	-0.89058090034680
C	-0.27500077048754	2.24334934071868	0.53679756128074
O	0.19126181725925	3.24480823901049	0.87514270365610

$(CO)_4$ Ring

M=1

O	-0.488747	-0.972531	1.774869
C	-1.001639	0.167679	2.169150
C	0.154223	0.883853	2.900848
O	0.100909	1.980673	3.361483
C	1.355722	-0.042039	2.907542
O	2.434839	0.089065	3.388686
O	1.396319	-2.259725	1.822260
C	0.883382	-1.256944	2.130653

Supplementary Material

$(CO)_4$ Ring

M=3

O	-0.493289	-1.015659	1.761463
C	-0.864428	0.164778	2.195226
C	0.116176	0.921017	2.897125
O	0.113188	2.020873	3.402557
C	1.318353	-0.036634	2.891148
O	2.385114	0.106981	3.374319
O	1.403261	-2.279476	1.819776
C	0.862035	-1.291117	2.112208

$(CO)_4$ Linear

M=1

O	0.96288057301979	1.38161523388068	-0.02268954716025
C	0.40124895332468	0.40137550406796	-0.01424680362168
C	-0.52914629404831	-0.69076049557291	0.00116914464161
O	-1.73755402427023	-0.38889372396998	0.01938382311573
C	0.14477067096496	-1.94442479268791	-0.00719747516479
O	1.35128843240030	-2.25123870908590	-0.02561770719750
C	-0.78937440392993	-3.03520055975465	0.00982237629591
O	-1.34996004295923	-4.01578467008246	0.01945484082895

Supplementary Material

$(CO)_4$ Linear

M=3

O	1.02207786620749	1.35458387055863	-0.02313167357529
C	0.36838689303698	0.41773810418510	-0.01455539963223
C	-0.51934366070183	-0.65605015188094	0.00209641378317
O	-1.74980173918950	-0.47497303403317	0.01902886866367
C	0.13436080058735	-1.97981676648950	-0.00531523515141
O	1.36411870459429	-2.16387298995279	-0.02661424129455
C	-0.75651415950331	-3.05176375184746	0.00699741654889
O	-1.40921215219389	-3.98927533403419	0.02152921907608

$H(CO)_4$ Ring

M=2

O	-0.00311255819753	1.05258526855334	3.16757217510265
C	-0.71895288192457	0.50251118200036	2.17203233642740
C	-0.22730646496151	-0.72688896977581	1.68970313717847
O	-0.68541601483818	-1.42767804950638	0.79540859062455
C	1.00012034646409	-0.98325833642376	2.52481642067404
O	1.77481166796488	-1.88390842776853	2.51508496185065
O	1.86471519136037	0.50771242865892	4.30431739454389
C	1.08916450932035	0.22800267118744	3.47034675854790
H	-1.58343599961248	1.05233965123776	1.84565869358428

Supplementary Material

$H_2(CO)_4$ Ring (1,4-epoxy-1,2,3-butanone)

M=2

O	0.05366491658095	1.02815396293822	3.17462165250473
C	-0.78242955098134	0.53668613198796	2.12150746268523
C	-0.19682107698631	-0.77475526306195	1.67344230988902
O	-0.60490062564700	-1.49630529787634	0.81801735677855
C	1.03515526404209	-0.99146854148643	2.54850352447010
O	1.79700764632666	-1.90226838796180	2.52763625881502
O	1.89001822844104	0.45804325109484	4.31532916163754
C	1.09333454696678	0.22183800431740	3.47211504757802
H	-1.79324113845582	0.40791813797121	2.49778989705527
H	-0.79801390633182	1.25827610979542	1.30991038768381

Compound $[SiO]_m$-SiO-(CO-COH)

M=1

Si	-3.05228961556117	1.74331755740195	2.92691837180852
O	-2.06620913487749	0.55682676278516	2.23585299657478
O	-3.23558291502987	-2.05566342517031	2.58822657781297
O	-4.52254105451046	1.27526957828440	2.45664792963323
Si	-4.83169627350415	-2.42278139345599	2.65125062255592
O	-5.71021827989945	-1.07561715976042	2.42768379522529
Si	-5.89744809010973	0.48515485490120	2.82410044688840
O	-5.10602580300702	-3.06032983375435	4.11084335676785

Supplementary Material

H	-5.19841160701484	-3.39859893522232	1.63364415811491
H	-6.99473239505117	1.05546464808091	2.05862931445396
O	-6.19876369378088	0.62998809689152	4.40568463059881
O	-1.42742834663948	-2.77546050193949	4.71320241941371
H	-2.73419459798901	3.05428895872778	2.38938769727731
O	-2.90340923550986	1.69933373463041	4.52231693186888
O	-3.60955437842466	-2.62375508985927	6.26518134175342
Si	-5.11825712203545	-2.79966178517333	5.71758261789996
Si	-2.09253299963995	-2.08383397804570	6.01279098399632
O	-5.96343228610011	-1.44651226898322	6.03054716637108
O	-2.16924960454935	-0.46334970328529	5.82996831998954
O	-4.47497750073507	0.72297351785342	6.40638930578807
Si	-5.92711268740238	0.17049961129510	5.94101193049147
Si	-2.92886745316331	0.95162855794750	5.96788457932141
H	-1.26725932278404	-2.41444146405117	7.16704118042668
H	-2.27511182015039	1.80287673909276	6.95064815537296
H	-6.95873826387539	0.72669209048454	6.80294040574282
H	-5.76058346499295	-3.92911535019684	6.37242237425100
O	-0.18563336705176	1.62222691331013	2.83321536311247
C	-0.73102740306792	0.68871974086798	2.31511054750995
H	-2.94127272577690	-1.22742916102728	2.19957999980561
H	-1.90043024161587	-2.66395130693469	3.88098368250488
C	0.01101074478317	-0.32622787090669	1.53332478938617

O 0.43859140658825 -1.25576934830822 2.31713093522315

H 0.96185953247863 -1.88876278648015 1.81370707205847

Compound $[SiO]_m$-SiO-(CO-CHO)

M=1

Si -3.08098586825360 1.81017857772663 3.00279775033173

O -2.14305648601033 0.54939935456408 2.41275410740101

O -3.27464650737160 -2.41344906576386 2.31941966395639

O -4.56785659271409 1.41351442471682 2.52633488789057

Si -4.88938407388032 -2.49139227860510 2.55052705905360

O -5.56679703251842 -1.03179658514496 2.34749383842153

Si -5.87192540290570 0.48522435010444 2.82320770284242

O -5.07800894246315 -3.01422703329882 4.06842164883961

H -5.53063299063814 -3.42232958852395 1.63032013929448

H -6.99968792786772 1.01407933093623 2.07165263146711

O -6.20870798693323 0.51234729873005 4.40467261301595

O -1.34639821700663 -1.96811100026713 4.29100606368068

H -2.66802682403288 3.06484506851248 2.39445216886923

O -2.96387466854321 1.87452613622611 4.60023574766250

O -3.28721213039111 -2.61839577656481 5.98882750276399

Si -4.86279486940879 -2.78139237586197 5.66082555521685

Si -1.91497292960960 -1.76392739960284 5.79659351034607

O -5.66291476798320 -1.44161023409394 6.10326729466349

O -2.23448711147323 -0.19586711736062 6.07711091689812

O -4.59103095061862 0.95350129328307 6.45018441947656

Si -5.91102445317832 0.15054981066579 5.9589199197245

Si -3.03076477029057 1.20446406029034 6.08110823620116

H -0.91350586555930 -2.20425625408585 6.75801167601512

H -2.44788449299581 2.12242264216688 7.04686770871349

H -7.04858585250641 0.54395734937299 6.77603737216122

H -5.39342026407391 -3.92523968170393 6.38633802106226

O -0.18792784945927 1.20075153974784 3.30111524719167

C -0.84407846350234 0.44183063478086 2.64955640332215

H -2.86425628831878 -1.91277432866150 1.61040897069841

H -1.97951277935480 -2.24864941874212 3.61347085337759

C -0.20668588647690 -0.75274068115217 1.95751777558279

O -0.79628667869881 -1.45466502110394 1.19152203822623

H 0.85236592503883 -0.88262803128711 2.20748055563143

Compound $[SiO]_m$-SiO-(COH-CO)

M=1

Si -3.00540149366388 1.65870119962558 2.89374483161174

O -2.09890628064547 0.38224251315079 2.33723954007034

O -3.18005655869045 -2.16377897826700 2.61918957106919

O -4.50870756162194 1.27122832623370 2.45438651651439

Si -4.78308744054804 -2.49072912808691 2.68507662945720

Supplementary Material

O	-5.61352228018765	-1.13234593818735	2.36270534768341
Si	-5.86417670384012	0.42184070821364	2.75456028163540
O	-5.10611796852370	-3.02952033473835	4.17820261053980
H	-5.15938170319783	-3.52141723518563	1.72780278463988
H	-6.94715448012461	0.95725508575249	1.94534793964525
O	-6.24110349351286	0.53732141549622	4.32351641467045
O	-1.27957914126440	-2.16133978375557	4.65189200156239
H	-2.56684450121858	2.89632346997238	2.26374038047512
O	-2.87863102823388	1.78415486197337	4.49219784217205
O	-3.42587468910045	-2.58158623189379	6.17533112098236
Si	-4.98722105865269	-2.72748055926852	5.76974373982193
Si	-2.01060179414354	-1.79827448166982	6.06621424114848
O	-5.77146309852314	-1.35169089851821	6.12167865133280
O	-2.26797988588018	-0.19916541987352	6.14507358639898
O	-4.60127936486271	1.01632984306607	6.33993629832745
Si	-5.95370801664424	0.24190886813137	5.89349414774016
Si	-3.02584647628811	1.21832876113194	6.00825864761677
H	-1.12919185561715	-2.19214150173090	7.15604206967701
H	-2.45553409150730	2.18516367554158	6.93287513349638
H	-7.07298531100646	0.71973146463260	6.69003384042889
H	-5.58202826472235	-3.82882653911037	6.51071000961041
O	-0.14887888866120	0.23205921661086	3.65316829817010
C	-0.72150457917323	0.33768131979280	2.43529921875184

H	-2.88775238851238	-1.30306321874286	2.28892211376535
H	-1.87365490614785	-2.36456664629355	3.91242103363191
C	-0.00152021386617	0.39458903091623	1.33795817866977
O	0.64341063723089	0.46542668370108	0.37524522760547
H	-0.33322511864834	-0.65180954862033	4.01317175107732

Compound $[SiO]_m$-SiO-(CHO)

M=1

Si	-3.16920335781642	1.85081531936552	2.90931857694153
O	-2.33987241244121	0.54693690731509	2.21425164553579
O	-3.51350064608287	-2.05600730001592	2.14189082221351
O	-4.70820552848362	1.49670094357775	2.58184667584606
Si	-5.09667919116610	-2.30127946408041	2.48236484420534
O	-5.86168264984735	-0.87006260658556	2.47287311082868
Si	-6.03883504312702	0.65280274900002	2.99250101869562
O	-5.20268668363429	-2.99838943582770	3.93849243465292
H	-5.70310221522232	-3.18814488090261	1.49927840538451
H	-7.19859558030431	1.25597635675095	2.35513380437667
O	-6.22249743654462	0.67648922570570	4.59928449024881
O	-1.42396071976024	-2.57922303693570	4.00628775129333
H	-2.79262459765077	3.10243084468739	2.27648032056154
O	-2.89785248685538	1.87583375586795	4.49290808661430
O	-3.37711587288580	-2.72539634799720	5.84776579301822

Si -4.95976453555379 -2.83292418720801 5.53735824998478

Si -1.94180309377228 -2.06533811758813 5.44976290658941

O -5.71177438717584 -1.48494668483910 6.04048190926966

O -2.13743517674058 -0.44677754247828 5.42876058262761

O -4.30021561089292 0.72730569018590 6.41838394337074

Si -5.76718929980643 0.13229950975747 6.06176415954646

Si -2.80304278886267 0.96355989818036 5.83957331221825

H -0.94457819289591 -2.42906785546050 6.44741681160741

H -2.01114921504048 1.66422626045395 6.83872271362011

H -6.72819870797167 0.57481954521948 7.06004986301841

H -5.51851838054563 -3.98564826010203 6.22710730438721

O -0.35648680506346 1.40067224584691 2.77582588485740

C -0.99945161466415 0.51504747479775 2.30740348279194

H -3.22305817180324 -1.15920603336781 1.94538036402970

H -2.06473267373473 -2.59191851603313 3.28531910685534

H -0.58982692365393 -0.41136645729007 1.90325162480871

Compound $[SiO]_m$-SiO-$[H(CO)_4]$

M=1

Si -2.92303603901901 2.18915128406093 3.47658535261373

O -1.43396178604177 1.50902739619637 3.41924688573191

O -2.76565467782615 -3.09214161378587 2.47170645789441

O -4.00338461097264 1.27045513574666 2.70916854079416

Supplementary Material

Si	-4.36886756927801	-2.82887216712999	2.67091413026774
O	-4.70866321514349	-1.26528999057277	2.43817996867259
Si	-5.24725153419797	0.23101906045194	2.71873615771409
O	-4.66704574815028	-3.25083025515451	4.20173399682778
H	-5.16583210929135	-3.63956863704880	1.75653758492963
H	-6.19230979196899	0.61548112205662	1.68004901101339
O	-5.97289974272664	0.28985514375412	4.16482691272910
O	-1.54911284266222	-1.44658872859691	4.36466114978114
H	-2.89397217533854	3.51074201372389	2.85960024976240
O	-3.26385866886665	2.29854812238100	5.04864159019712
O	-3.23598680676337	-2.31694899626821	6.23006298882439
Si	-4.70247114545789	-2.82748163879555	5.76863241499340
Si	-1.94504946699360	-1.37098015600496	5.94646290945654
O	-5.77492069767423	-1.62841526309146	5.97552572066594
O	-2.30158044180507	0.16979283275581	6.29411991296289
O	-4.87085860450876	0.79880866343010	6.52055298901419
Si	-6.02507595191879	-0.04319023059363	5.75195436530190
Si	-3.36310517117223	1.38592785644256	6.39426169409748
H	-0.84472989734731	-1.79788612490949	6.80055536001058
H	-3.07043324971049	2.21429192690460	7.55345059082506
H	-7.33131271452489	0.31785627671043	6.28022874208151
H	-5.09916582582093	-3.97879175212013	6.56377471906156
O	0.69355468250739	0.86291397510810	3.03454445716300

C	-0.58109216727991	1.10455524736860	2.43980372036685
H	-2.33169182445513	-2.82487463184469	1.65970716518130
H	-1.87257955713936	-2.18898245494334	3.83346885405618
C	-0.97998776391481	-0.22654680908481	1.81258505492910
O	-1.93004222285005	-0.44092353612299	1.12538475100602
C	0.10148979444484	-1.22154547650980	2.19528170005658
O	0.17922078107968	-2.35712492092786	1.85076782410548
O	2.08456631853892	-0.81134947816362	3.55296242449130
C	1.09543662551822	-0.42930890871074	3.03036076344657
H	-0.43704418126819	1.86663571328841	1.67351288897245

Compound $[SiO]_m$-SiO-$[CO(CO)_4]$

M=2

Si	-3.13275051882750	1.87807532356208	3.03591430982266
O	-2.06689659753577	0.75635093814329	2.42457672518246
O	-3.26164191439144	-2.18530340527094	2.25018500056317
O	-4.56540807912060	1.33135202108131	2.54419026960525
Si	-4.85140795939755	-2.45706087703977	2.55071108875013
O	-5.67820531392771	-1.06586941414431	2.46556994400597
Si	-5.89599784560195	0.47308860084853	2.92283026391146
O	-4.89094048862441	-3.07174763702316	4.04274517666965
H	-5.43486419766442	-3.39890889356868	1.60462148116885
H	-7.03432865598274	1.03254153865306	2.21111369246957

Supplementary Material

O	-6.14877758135063	0.54535587200843	4.51761698076440
O	-1.29830550508014	-1.79879540231237	4.17473998855650
H	-2.85772621728714	3.19562773590761	2.48081119837418
O	-3.01860242954421	1.91561923736494	4.63607510160154
O	-3.14579359953818	-2.50437063552048	5.95323993751955
Si	-4.70332938228667	-2.80508948315635	5.63568295023689
Si	-1.77550174378076	-1.66162070474448	5.72825712607270
O	-5.60826396504368	-1.53014665205360	6.06580691268025
O	-2.03556740161560	-0.09571274173042	6.06812512369953
O	-4.46591114327390	0.81703923117425	6.53800221574566
Si	-5.81783022183656	0.07655525900286	6.03732167033245
Si	-2.95597942332767	1.22761239073334	6.11080615080319
H	-0.72426954692109	-2.16036337270065	6.60032954311627
H	-2.41908943108068	2.18792853336984	7.06072243501949
H	-6.92439715002190	0.44003410068965	6.90828669406941
H	-5.14022920965759	-3.98172616616178	6.36971257591809
O	-0.17568426413199	1.06039954610395	3.71641595588089
C	-0.72159026594610	0.76294225967344	2.44017066771775
H	-2.97585376880486	-1.68224396530875	1.48774730353864
H	-1.96195314474100	-2.03203495777626	3.50644075410588
C	-0.13338282965342	-0.56734774653567	2.01319119640774
O	-0.49087513395673	-1.22203925977061	1.08454068412511
C	0.96582003967541	-0.86487499701763	3.01978335105171

O 1.80741716375963 -1.69752120041781 2.96788845516819

O 1.28682488557691 0.22630586967282 5.18843927417198

C 0.76573393109791 0.16146814094876 4.13229528256412

C -0.18332099543807 1.93673906238090 1.51250396154317

O 0.62415990528348 1.88864185093478 0.68045855706477

Compound 1

M=2

Si -3.76374491486483 0.14100377748388 3.76098904442969

O -2.98698629594247 -1.07674684128106 3.01071513889314

Si -2.63171905144912 -2.65272802916670 3.08036999569077

O -3.97238018947339 -3.52853536812111 2.79549474227080

O -5.35129951001588 -0.20790699626023 3.82471459944353

Si -5.42899341391241 -3.98331931236935 3.33263706052249

O -6.33025866540049 -2.69109522995254 3.69924165912448

Si -6.44984242577731 -1.23722831757550 4.41923361860006

O -5.24418900324510 -4.88980676010846 4.67966476502913

H -6.09554737871525 -4.78544108091283 2.31923362636536

H -7.77986404895766 -0.69993622406059 4.16275994596151

O -6.15224298600472 -1.40535699847048 6.01590779641378

O -2.06069972670132 -3.02321726666670 4.55365771443175

H -1.63055520118849 -2.97362842367244 2.07525596959800

H -3.57692147818597 1.36332688471570 2.99415173007065

O -3.16899971074201 0.32338617262595 5.25047198927990

O -3.31275912013378 -4.32599743746160 6.47635201570487

Si -4.76273351337196 -4.96915647096126 6.21448693500809

Si -2.22568544811859 -3.14526405462016 6.15888833653165

O -5.76855413261380 -3.98159960158019 7.12346128351935

O -2.75856155933797 -1.76867865430740 6.78952781163834

O -4.39762242468415 0.16759218878280 7.63905046049307

Si -3.05753357866637 -0.16082222390200 6.80183850960739

H -0.95370160074792 -3.54071618165056 6.74625354464582

H -1.94855273664339 0.53776957459097 7.43855296550136

H -4.82094225702457 -6.35459116652325 6.65858537265397

H -6.18444581533515 -2.27864635817561 6.42692251244338

H -5.20682381274571 -0.22439960039914 7.29476085612738

Compound 2

M=2

Si -2.98696147743679 1.79168737351597 3.07921032984725

O -1.92095950900977 0.60055500163040 2.50315583975199

O -3.07349886243041 -2.09619184357458 2.50582894332340

O -4.40881742468846 1.30052772387326 2.50845510109194

Si -4.67252644489984 -2.44366011868162 2.59222078153556

O -5.52740432348683 -1.08673604142081 2.32546807133955

Si -5.78226465993599 0.45382798221845 2.75606916429116

O	-4.94390596324114	-3.02930325327906	4.07208293826884
H	-5.05812755096687	-3.44348658984335	1.60638254075951
H	-6.83658988404095	1.02268441698760	1.93230185200311
O	-6.18515498551573	0.54316154666176	4.31644174082151
O	-1.30683751246941	-2.34939974874466	4.69953354529139
H	-2.60707344161232	3.07776154055473	2.51682542650350
O	-2.94031177276583	1.82367549483099	4.67810575683076
O	-3.47928722736829	-2.59755018884951	6.24667328477083
Si	-4.97829827569437	-2.79884096707906	5.68269597128981
Si	-2.01809013854094	-1.89381384229028	6.07200080690527
O	-5.87098983505625	-1.47709179126630	5.99968945075831
O	-2.25191475088467	-0.27964447206350	6.10082325145412
O	-4.63786790145226	0.83201687224210	6.43626763435209
Si	-5.98933349182692	0.13158651574963	5.87749734383186
Si	-3.06632613015373	1.10392112921737	6.13917264953945
H	-1.15872793259969	-2.26872790676169	7.18606569183110
H	-2.53253379909176	2.00491860657052	7.14857930825446
H	-7.13260833716409	0.59715518610293	6.64657755218369
H	-5.59402735638867	-3.95543528135096	6.31450466750301
O	0.00745064019089	1.26903707118571	3.48381999112161
C	-0.63598948434207	0.54682855602522	2.81509787773795
H	-2.77735461494440	-1.28619555132698	2.08756302574727
H	-1.82550755218244	-2.37277742083428	3.88701946105966

Supplementary Material

TS_I

M=2

Si	-2.89720597058741	1.77759760016296	3.22479799600532
O	-2.02556058229915	0.49538419610770	2.54021694626728
O	-2.89264177034926	-2.62394220022058	2.63309010307222
O	-4.41157909669365	1.48646557270269	2.76861081978433
Si	-4.52497066243990	-2.55738394863808	2.65278443022177
O	-5.02829397187304	-1.04749021143459	2.33822167145869
Si	-5.62695460142794	0.39859084988287	2.74632504861700
O	-4.96503831525422	-3.00787631706229	4.13892231326279
H	-5.11350169021948	-3.45945717496187	1.66989593922113
H	-6.61208765191528	0.82841277102008	1.76632988110618
O	-6.30544542045508	0.32639700067833	4.21232188191710
O	-1.36538319720941	-1.75509887310894	4.79873054380439
H	-2.41361869788716	3.03008829796836	2.66604701885287
O	-2.71209499013097	1.74344382693374	4.81557518810209
O	-3.42481229273651	-2.57899072112104	6.25786117726056
Si	-4.94496128051212	-2.77639189203243	5.74863873039452
Si	-2.05475955310426	-1.69692629518639	6.26106776549112
O	-5.81564176990131	-1.45250352500412	6.10837428230638
O	-2.42378028015081	-0.15687077650179	6.64627559926363
O	-4.78404990899797	0.98997659581393	6.26455615543039
Si	-6.07091611496965	0.11727699935315	5.80692385373387

Si	-3.17891485224595	1.21973832279051	6.29043762722320
H	-1.15403635703054	-2.19723179300061	7.29091107117689
H	-2.84893265687116	2.24250136181543	7.27046701217767
H	-7.25057342677731	0.56761520088481	6.52869759497820
H	-5.53862624707632	-3.93253646862017	6.40194398366182
O	0.10760290549045	0.99548687983664	3.12163226254134
C	-0.73378502035552	0.34631655035273	2.62093750096610
H	-2.37027516371423	-2.19375977699943	1.95073184259247
H	-1.85175175925036	-2.17880946119707	4.07906767424263
C	-0.26442257295056	-1.47140619769804	1.54027346342237
O	0.84145296989609	-1.57875639351650	1.31648262144368

Compound 3

M=2

Si	-2.91860598361132	1.91582748784712	3.25962352199817
O	-1.98019549929573	0.84212613966804	2.37872450762634
O	-3.13916525117869	-2.85693049786823	2.35492452987516
O	-4.42254811064793	1.56240681002725	2.79828777033598
Si	-4.73309478441521	-2.54678316984774	2.58237575439485
O	-5.03798974602032	-0.97338480041301	2.34509025197519
Si	-5.62414025747387	0.46795966823484	2.80234843557880
O	-5.03094462007732	-2.97372612050034	4.10651019325883
H	-5.55831249787869	-3.32896300661121	1.66706837606491
H	-6.64979677621298	0.90649773358729	1.86765053815022

Supplementary Material

O	-6.25957115315800	0.35641081301007	4.28600507655960
O	-1.79035817338051	-1.49787932664400	4.42260916649770
H	-2.58040771332049	3.27699135259454	2.87293547659937
O	-2.70365752750792	1.69855022632890	4.83169781447651
O	-3.43216914715769	-2.62227373951647	6.19074237126287
Si	-4.97096731944036	-2.79327475933876	5.72082357396848
Si	-2.09274797288606	-1.71194471528730	6.00165110665846
O	-5.82630878994046	-1.47846193350365	6.13899935284079
O	-2.32821867780751	-0.22862153468298	6.62779464429377
O	-4.70532485789918	0.91817400419254	6.34968735768888
Si	-6.02348653228362	0.10790858899210	5.87421365983251
Si	-3.10059901807026	1.15559469273430	6.31229285191517
H	-1.00283966334700	-2.35051569793234	6.72917031493325
H	-2.73437846458458	2.15305533374769	7.30568241360090
H	-7.18308951934244	0.58007055844625	6.61499863462025
H	-5.55166564854828	-3.96675366458670	6.35503431050093
O	0.03666400033902	1.09558821334160	3.36707520693418
C	-0.70425773902313	0.56268347963019	2.60202004093161
H	-2.71830737717698	-2.66021251488377	1.52063629137148
H	-2.07714637254768	-2.14272034943260	3.76659872404883
C	-0.23264169473603	-0.44318920053591	1.59217739251329
O	-0.19427711136869	-1.60973007079770	1.58795033869262

Supplementary Material

Compound 4

M=2

Si	-2.97507384978623	1.92702164958669	3.06005121901050
O	-1.73189835566675	1.15317641343776	2.26973162257424
O	-3.52805613468978	-3.07254960831497	2.12925922122311
O	-4.30904112076926	1.18521219442164	2.55515663925820
Si	-5.06330472981289	-2.77033658718469	2.63432232998633
O	-5.43122438874661	-1.21751496089831	2.37032629937052
Si	-5.66258406435220	0.32594973950447	2.80054816667547
O	-5.07563255905419	-3.12659069462199	4.20393806601970
H	-6.01779735554365	-3.60539217978385	1.91177982055897
H	-6.72336699116738	0.88588159455610	1.97549067373999
O	-6.08583388594869	0.41454381147116	4.35730355542620
O	-2.34736700260751	-1.28060120495588	3.92095153603758
H	-2.98128259992738	3.31631998982055	2.62405946773564
O	-2.77437936586002	1.86778068780147	4.65039545664246
O	-3.26473374821690	-2.55324590104447	6.06462564875183
Si	-4.84201036134167	-2.79847982417729	5.78012145249495
Si	-2.05591187825779	-1.62096665190997	5.48745599646197
O	-5.69077848520796	-1.47103264397859	6.17227909259272
O	-2.06050917404716	-0.17131165563776	6.21862788509323
O	-4.48041898599696	0.88319376782226	6.40291029984252
Si	-5.83212894138571	0.12572551467384	5.93418020192526
Si	-2.91232936099220	1.20073402905062	6.12888796613845
H	-0.78988300950959	-2.29668825578813	5.74062960409903

H	-2.42730855378726	2.14538524953178	7.12220237648561
H	-6.96247654150087	0.62352395167970	6.70304637173066
H	-5.31296046396895	-3.92409178018056	6.57236610228354
O	0.04233333069300	0.88811818986778	3.90746815368403
C	-0.46747291631772	1.00316801721650	2.55470015010869
H	-3.32840465579930	-3.02978646333256	1.19691815909890
H	-2.48863121789483	-1.98295143816901	3.27631077082528
C	0.31969980045795	-0.08653787790986	2.97294246598394
O	0.92148756700645	-1.09330707255442	2.94342322814042

TS_IIa

M=2

Si	-2.86612852474655	1.83506622098944	3.22824311389167
O	-2.01698460665273	0.56574642380498	2.51525976713889
O	-3.10126672063925	-2.75395131350567	2.50947579608491
O	-4.37839255584308	1.60253247068040	2.71908250513685
Si	-4.71415922028955	-2.49820967097929	2.57595881100015
O	-5.04502355185103	-0.92420559062600	2.36971513086961
Si	-5.60755243691898	0.53637258812658	2.78288355166430
O	-5.16278672438265	-2.99458659802044	4.04524991801130
H	-5.42373113472942	-3.25982546120088	1.55399070567174
H	-6.63829333240277	0.97052564961780	1.85232415294702
O	-6.21300005111230	0.49277701177351	4.28141358374683
O	-1.41611593029346	-2.00956770174492	4.61266319122821

Supplementary Material

H	-2.34258634401170	3.09937462694909	2.73559916203231
O	-2.75844299714497	1.74736661952025	4.82280295390064
O	-3.54728756454288	-2.55242963360415	6.10477656419699
Si	-5.08196909932434	-2.77088742075953	5.65439375263527
Si	-2.07718729877803	-1.85154526466702	6.08041327021140
O	-5.95888690977911	-1.46091955825792	6.04878056454148
O	-2.23708359914855	-0.27442179698560	6.45218381478069
O	-4.65299795709803	0.81948605674651	6.39646295771666
Si	-6.02414235991657	0.14701990457835	5.85858819474481
Si	-3.05952110637823	1.09952630249477	6.28498307683122
H	-1.23066738722571	-2.45793931172899	7.10005797101664
H	-2.65665686217256	2.04770896518208	7.31205672935280
H	-7.15392794787715	0.67675329779166	6.60620783831517
H	-5.63846054368995	-3.93776258615142	6.32148584429387
O	-0.00823794585250	1.00944118833466	3.42922316693417
C	-0.71691207379239	0.37833156838654	2.70383820679812
H	-2.56342433652865	-2.42572985032950	1.78736527145835
H	-1.95619001670534	-2.38585388052690	3.90506289114123
C	-0.21262439084412	-0.62745329412152	1.70398169683402
O	-0.62293986446567	-1.67256738028569	1.35826408181025
C	1.83488588629659	-0.46481984666530	2.12335172754427
O	2.70792550884168	0.05107726518412	1.63556003551806

Supplementary Material

TS_IIb

M=2

Si	-2.91981665625444	1.77128942354557	3.15567319912991
O	-2.00058170008100	0.50382779724214	2.52017564775760
O	-3.00939327512050	-2.65476835594453	2.50134533795410
O	-4.41702198722427	1.42527563665282	2.67902048537759
Si	-4.63847446685109	-2.59170201051415	2.63864601973534
O	-5.15667605846712	-1.08354092254343	2.34568342490944
Si	-5.67419153438402	0.39320837433211	2.75455886736234
O	-4.96505406196625	-3.03436353812820	4.15452467526012
H	-5.29565471587351	-3.49853073938763	1.70451706269655
H	-6.69567030208235	0.84180364661446	1.82132365018990
O	-6.27680046635569	0.37416493004444	4.25505036235207
O	-1.42201823360641	-1.79064520390633	4.63271743416393
H	-2.47566554481162	3.02867422672604	2.57828649233211
O	-2.77439025603335	1.77135360517425	4.75013639797633
O	-3.40074842694111	-2.54975994509029	6.23538014008852
Si	-4.92784799977573	-2.77764627032717	5.75911474428726
Si	-2.01156472945171	-1.70403822522922	6.13852009264672
O	-5.81736521052117	-1.46415786638010	6.10709585507365
O	-2.30200540686960	-0.14764899819383	6.52277833625477
O	-4.71603852785391	0.93606068477442	6.31351472534400
Si	-6.03191560452983	0.11892139732329	5.84159898946177

Si	-3.12029348611382	1.21186054157680	6.24091435332681
H	-1.06040315516021	-2.22557037371443	7.11047077074766
H	-2.75374850744980	2.22323351382938	7.21929762041164
H	-7.19225946483960	0.58367121022269	6.58526563363436
H	-5.49206752957804	-3.93264758561306	6.44009068313718
O	-0.00738591856481	1.26615381981385	3.23006111926834
C	-0.69344751631830	0.44578308717725	2.70197495240927
H	-2.57908335043334	-2.26137264416553	1.73401136137990
H	-1.94147282052802	-2.22950881493073	3.94564907085228
C	-0.07331592860636	-0.73036816825885	1.98284221856899
O	0.73357676661935	-1.50072234135551	2.31455604189364
C	-1.21686426513298	-1.22000859701435	0.42742799490781
O	-0.96902965883938	-0.85582129435219	-0.62276376089190

Compound 5

M=2

Si	-2.87271413020278	1.86012583101171	3.15765531635265
O	-1.71848870421151	0.87262113238118	2.45561711434891
O	-3.53388283101724	-3.23644995409633	2.43311277125854
O	-4.26636837130427	1.25038762765315	2.62428989147678
Si	-5.08575004028405	-2.76944236937369	2.70406830392884
O	-5.24240539318117	-1.18793916264711	2.39664396130309
Si	-5.59929006544006	0.34456224144987	2.78628123995584

Supplementary Material

O	-5.35815429954867	-3.09475009889682	4.25773993994767
H	-6.01188296872608	-3.51295290921266	1.85607581392528
H	-6.62368542572880	0.85004168806259	1.88394053587354
O	-6.13735874116914	0.41420406233893	4.30917490066655
O	-1.85987866995865	-1.84957796791170	4.23106396036229
H	-2.71425142598354	3.22969779318656	2.69632209652100
O	-2.75822915643746	1.77301978021138	4.75530887230799
O	-3.52290782122890	-2.69554055883299	6.13372411710731
Si	-5.11135577694531	-2.79425844500153	5.83765648786547
Si	-2.15303275495574	-1.86536482789965	5.82950346770415
O	-5.83734821192596	-1.39578422402279	6.21996444740467
O	-2.31284236290554	-0.32636060968545	6.31803743692794
O	-4.62874124733745	0.95421786124257	6.41434815644685
Si	-5.96379889245370	0.18997407760659	5.90898698586707
Si	-3.02604796622854	1.11877052095364	6.21703770382936
H	-1.06653292870260	-2.47065853308247	6.5889098411731
H	-2.50128896086527	2.00490078328846	7.24304658705969
H	-7.12702113526404	0.72561772770777	6.59885238664960
H	-5.69142059027418	-3.87311734074286	6.62195632439965
O	0.08540542106755	1.90415678004616	3.31964196563886
C	-0.40595393880293	1.00401426794042	2.71470958858951
H	-3.17232120280050	-3.17326440043418	1.55138211828767
H	-2.33200821399130	-2.46311206003783	3.65526190712087
C	0.34277518734182	-0.14459659536136	2.13026641580666
O	1.57237543673638	-0.31552433376637	2.13561189714767

C	-0.47497434813380	-1.10342062939409	1.53953096497853
O	-1.05182946913644	-1.95125715468110	1.03722648176634

Compound 6

M=2

Si	-3.00557024866886	1.92091676056053	3.36002855045790
O	-2.10965136858498	0.78093569837354	2.50618903630673
O	-3.03025651822833	-2.35448273222800	2.30619784268058
O	-4.52201550785718	1.65208654916087	2.88694579573432
Si	-4.65661187966057	-2.33742456569976	2.39391752545280
O	-5.22742130955574	-0.82338913429167	2.26538853946130
Si	-5.74666468528332	0.57790905586558	2.88407633599775
O	-5.01015215965873	-2.95721809336161	3.84420235867440
H	-5.27363402109748	-3.14825564011712	1.35026439265410
H	-6.82566006639161	1.11248714877629	2.06785348811680
O	-6.26856592430817	0.35871083110381	4.39920270685439
O	-1.34584228660109	-1.83481690373682	4.44155286154276
H	-2.58541120911762	3.26016348618110	2.98229913162198
O	-2.81176698852310	1.66607940093836	4.92935556321678
O	-3.36670321200727	-2.73484420428200	5.91530271854663
Si	-4.90694383316148	-2.92391030503369	5.46266459253465
Si	-1.96846135627033	-1.90197571610422	5.93704360572183
O	-5.79521584351567	-1.68264363405347	6.01502909156331
O	-2.25225656679652	-0.39774490403094	6.48995839537082
O	-4.68226799843028	0.67405250438237	6.49104725406115

Si -6.00664413147286 -0.07915320871437 5.94016114183946

Si -3.09319773486868 0.97234351328346 6.37282355732488

H -1.03463557737891 -2.53900121780094 6.85680004757009

H -2.69172713570185 1.88811954995744 7.42935342575116

H -7.15378771931700 0.29820733501548 6.75159790458657

H -5.43112772051169 -4.16802564595479 6.00593259586625

O -0.09464366484294 1.29251421101545 3.36213152468080

C -0.81287548650966 0.61964759384055 2.66736749939369

H -2.51866901394022 -1.90732338169087 1.62071234629804

H -1.90790981819561 -2.13475543322669 3.71070347209708

C -0.23940569830076 -0.50655614841911 1.89021491653571

O -0.78847278504434 -1.28257274351151 1.08686483899928

C 1.11376893786691 -0.67542714479083 2.20639552833816

O 2.20464053193590 -0.92223288140642 2.40169141414792

TS_III

M=2

Si -0.98566301655566 -0.52609938862330 3.97219223611637

O 0.02921780221501 -1.86488649396623 4.08343451481058

O -3.71131263241571 -4.96883985448553 3.95025307419712

O -2.29904096499150 -0.92866500688994 3.15305520348460

Si -4.91652302805807 -3.93511508346601 3.54152818573963

O -4.31525420868931 -2.62766319507454 2.79522513491809

Si -3.87810085172868 -1.07309843690438 2.79427077452592

O -5.60658811654153 -3.53387793672714 4.94227010171893

H	-5.88493417351956	-4.57037426093204	2.65514982798358
H	-4.10501411011985	-0.51491990501401	1.46942083504324
O	-4.74365219549655	-0.25145982335287	3.88587394380144
O	-2.35384024928858	-3.15476081723382	5.59157194745952
H	-0.19438869723548	0.47018721778632	3.26950105394296
O	-1.27537912562101	-0.04569399811852	5.47273494382484
O	-4.40404344968143	-3.11747115217832	7.27679222911518
Si	-5.71598988016778	-2.78641219815514	6.37821237169541
Si	-2.79130203467468	-2.93857655070032	7.14756352566999
O	-5.81548231940045	-1.18528910043018	6.12492283299538
O	-2.35435618463080	-1.40937605325920	7.47961154160250
O	-3.74982833548474	0.47962389857860	6.22233860923285
Si	-5.11103556438147	0.09628660670634	5.42817516122767
Si	-2.28456667633903	0.03453028311517	6.75284935813085
H	-2.14504091259417	-3.84094451779079	8.09141451399667
H	-1.78035969939280	1.03121865236709	7.68359989527529
H	-6.02583527150421	1.22657286664255	5.46093516926502
H	-6.90807556308348	-3.24393398271666	7.07455936305133
O	-1.00227707106039	-3.57770583861535	2.95788375687135
C	-0.11425938153086	-3.09726109014368	3.79279572356923
H	-3.17939406112882	-5.38874718431402	3.27430496091593
H	-2.69268911214820	-3.91610142741229	5.10167490315338
C	0.55240366043754	-4.28670836986330	4.23093300448268
O	1.44600556241408	-4.60903756482895	4.99684161170530
C	-0.40339244475357	-5.02893690507144	3.37384207038341

O -0.87797769284805 -6.01939338892795 2.99610762009329

Compound 7

M=2

Si -0.93148366478145 -0.42436493645385 4.07607240176781

O 0.10684842056910 -1.74933330333294 4.13410154090113

O -3.72797881506202 -4.89829537899627 4.00442542303087

O -2.18888582211655 -0.79685934841126 3.16079073610599

Si -4.87278544498331 -3.82694116788425 3.52655712450777

O -4.17227770621322 -2.53137900253337 2.84850797941520

Si -3.77040048997028 -0.96889566518864 2.81926868183927

O -5.66168849690741 -3.41527620053149 4.87329826250783

H -5.79514324712096 -4.41717175920474 2.56287608149492

H -4.01162840178120 -0.42955373190557 1.48900611984957

O -4.63742498348109 -0.13558367935827 3.90044871907186

O -2.37964080033193 -3.07178509692876 5.67362356571595

H -0.12037511900191 0.62151670498310 3.47551217835135

O -1.30988962492169 -0.03799712904257 5.58069967818997

O -4.52535739615288 -3.04638677819061 7.24681601221794

Si -5.79675098447532 -2.67031023692343 6.30892913862142

Si -2.90722763667584 -2.87040550356153 7.20320139555812

O -5.83978371033959 -1.06457462011276 6.06808993772703

O -2.48106157590155 -1.34657042958398 7.57861836356423

O -3.77292274920035 0.59127855526395 6.29090615886593

Si -5.08939463619406 0.21506113474738 5.41946258681744

Si	-2.33452083308942	0.08841507616687	6.84474176788608
H	-2.31760028734667	-3.78689176386280	8.17121262440855
H	-1.80333606126350	1.06451027196144	7.78256224157219
H	-5.99502188564011	1.35325190805034	5.40613494432134
H	-7.02550960611588	-3.09420914650436	6.96169676705088
O	-1.04283557500577	-3.48538532517779	3.10249967559304
C	-0.00019317534257	-2.97262686060426	3.79750448713192
H	-3.15401526707217	-5.31533891740052	3.35966865595458
H	-2.70151037259334	-3.82430531845893	5.15957611121451
C	0.75695595305018	-4.16463143823907	3.96335017888401
O	1.80565695714193	-4.53282040700743	4.45402409860362
C	-0.40602227363073	-4.81887152099600	3.24632966325185
O	-0.91279468804844	-5.81527898477759	2.90017669800586

Compound 8

M=2

Si	-3.02290587407096	1.90004914949771	3.21926609928377
O	-2.08676035581971	0.62248283974366	2.64085302119052
O	-2.99759213487712	-2.47298958899041	2.42077252790429
O	-4.50469145793871	1.55117979847930	2.68875123422509
Si	-4.62567153028229	-2.46574745301135	2.51770461679418
O	-5.19568424940536	-0.96649401946846	2.27748296684026
Si	-5.72894995788766	0.48149108271418	2.76374077365024
O	-4.97291669598374	-2.99617594383366	4.00417975757346
H	-5.23542250149950	-3.35373276309042	1.53474199596467

Supplementary Material

H	-6.79528677501887	0.94078397425328	1.88718913967270
O	-6.27507285866635	0.38453085399930	4.28264896605833
O	-1.29798588140536	-1.91846780009651	4.56251480432587
H	-2.55725579722614	3.14994540007280	2.64183330199334
O	-2.95885547099898	1.93721137452026	4.82224292004687
O	-3.37648319810477	-2.58052215102202	6.07999123529435
Si	-4.90727078506144	-2.81568957314917	5.61654647385126
Si	-1.98853911530742	-1.73267242428051	6.01803401149969
O	-5.81553086259614	-1.53900597197353	6.04087522645057
O	-2.32081412261394	-0.16598687108398	6.29184607311888
O	-4.76228925886980	0.86206011132759	6.40534759833090
Si	-6.05377046167188	0.05227782008842	5.85706067256646
Si	-3.18240301239595	1.19435346038109	6.25213028663147
H	-1.07824084985869	-2.19240854392910	7.05821946310686
H	-2.76620706434780	2.09075220340733	7.31906088251664
H	-7.23350609150840	0.45785423133023	6.60529394593447
H	-5.43430228957442	-4.01172501113757	6.25558316166730
O	-0.17337129783380	1.41666627955969	3.48901507281678
C	-0.77797458981464	0.60515384766076	2.85509647382200
H	-2.51138813354909	-1.93826773565995	1.78483618178615
H	-1.86787175910436	-2.22648288172662	3.84186491776686
C	-0.09926445204877	-0.55400322601265	2.15130553969155
O	-0.69223921784958	-1.30188494604395	1.39266361279300
C	1.33033329661209	-0.79367547171967	2.34308338130102
O	2.23386980193126	-0.24425055365405	2.97874778227230

O	2.18888140613324	-2.83116359361783	1.06921666075684
C	1.69266359851504	-1.95304590353421	1.58205922050108

Compound 9

M=2

Si	-3.04657744816917	1.98444080118237	3.14474521818554
O	-2.13643018059796	0.78186354701523	2.36917165148594
O	-2.92876639407569	-2.36474755651832	2.41180130510174
O	-4.54461956008560	1.62342768038257	2.71283797515460
Si	-4.55232791569999	-2.40082711389484	2.51096586155069
O	-5.17002988622329	-0.91290175259538	2.28746512233446
Si	-5.74063234270198	0.50854124815036	2.79517403730574
O	-4.87638852380504	-2.91899888206112	4.00749440280838
H	-5.15571142407102	-3.29564005718161	1.53093779346285
H	-6.83198569688172	0.95398791899083	1.94421406903835
O	-6.24426645506093	0.39827228615668	4.32671132935281
O	-1.18041053804819	-1.90865938611360	4.55130234373540
H	-2.62667658289269	3.26587254529756	2.60464981628765
O	-2.78594843196848	1.89373560975325	4.71775446297807
O	-3.25447508124295	-2.57766282248771	6.07481346468839
Si	-4.79341009145741	-2.78094098968722	5.62077103968558
Si	-1.86756794485336	-1.73352960084135	6.00992572196505
O	-5.66917551986287	-1.49374273264144	6.08497733823487
O	-2.19745677426402	-0.16051162792004	6.27344673847045
O	-4.61633169824418	0.92439022824517	6.34167559524013

Supplementary Material

Si	-5.93320680204073	0.08973724029437	5.89113351731698
Si	-3.03155271407120	1.20974815406569	6.18364035970463
H	-0.95404391384806	-2.18343026241282	7.04993959040482
H	-2.59066253101984	2.14714745276552	7.20357919791632
H	-7.06814126139267	0.50307757238290	6.70078941969289
H	-5.33498501118575	-3.97948937659778	6.24150654553474
O	-0.21659024977606	0.86463236681942	3.52921195019355
C	-0.95611024544131	0.33291699465477	2.55706846354387
H	-2.44325534183583	-2.05837744416458	1.63101263103566
H	-1.74038256274563	-2.22470019507076	3.82640723254203
C	-0.34271820928046	-0.71384740558076	1.81712177656082
O	-0.86988458125315	-1.37133264741646	0.90652452080439
C	0.98432809335779	-0.87084086276221	2.43723615572639
O	1.89093023353656	-1.60570151060195	2.18309704211773
O	1.81750783358519	0.40840156098898	4.39357844671985
C	1.01457575361777	0.15081901940422	3.58340786311870

Figures

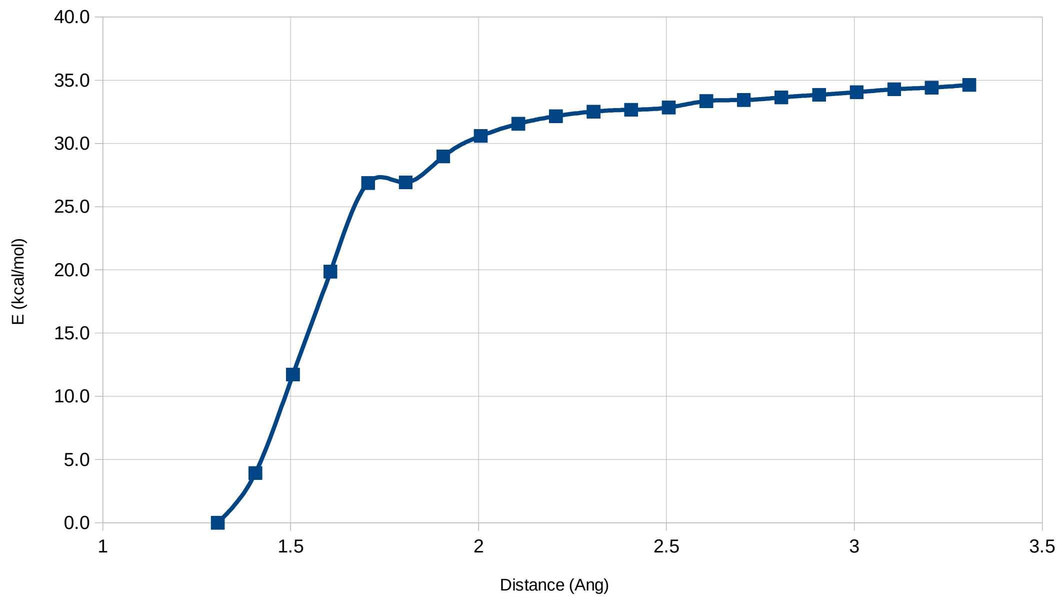

Supplementary Figure S1. PES of the reaction **SiO-------CO (compounds 1----->2)**

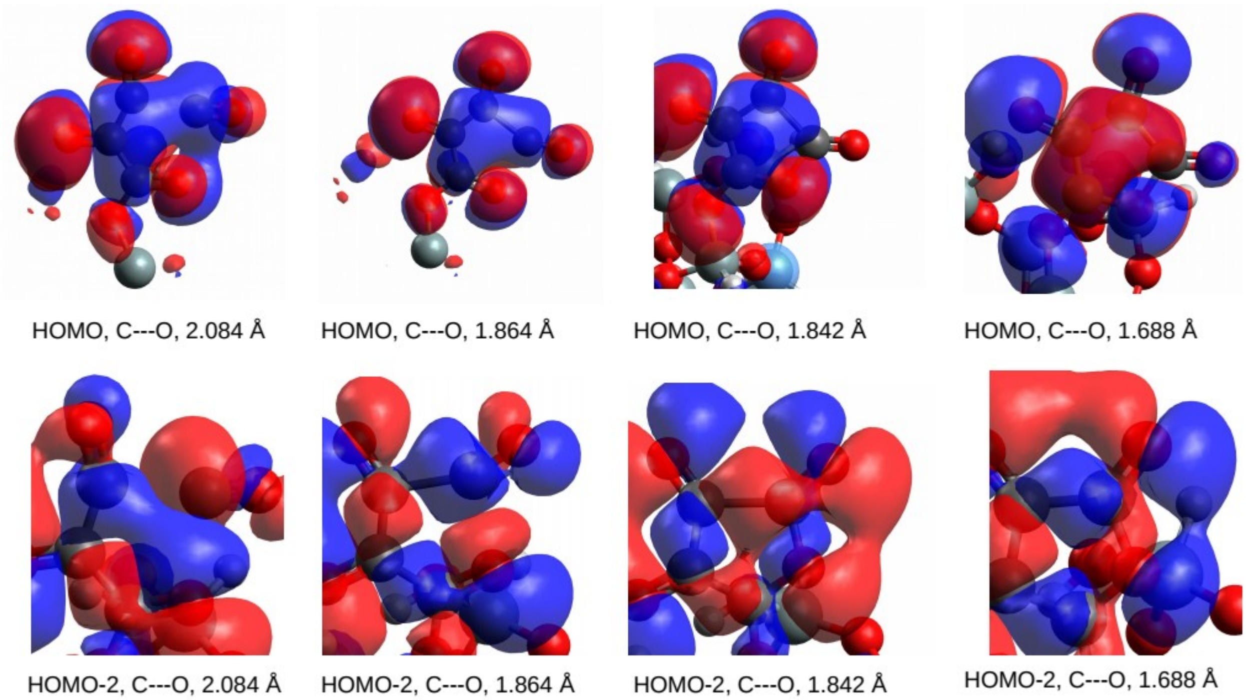

Supplementary Figure S2. Upper panel. PES of the reaction $SiO(CO)_3$-------CO (compounds 6→9). **Lower panel.** MO representation of the early radical delocalization on the $(CO)_3$-CO moiety (HOMO) and σ-bond (HOMO-2) “concerted” formation of the C-C and O-C bonds.

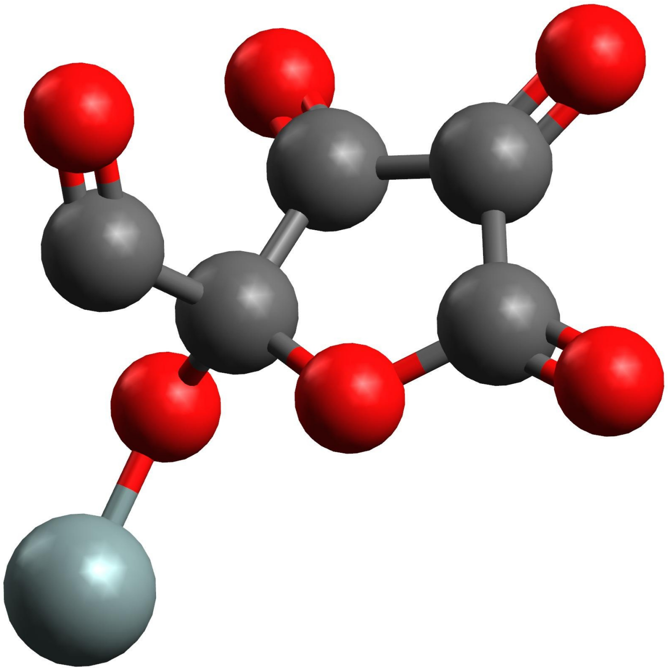

Supplementary Figure S3. The final product of the CO addition to the SiO-**C** carbon.

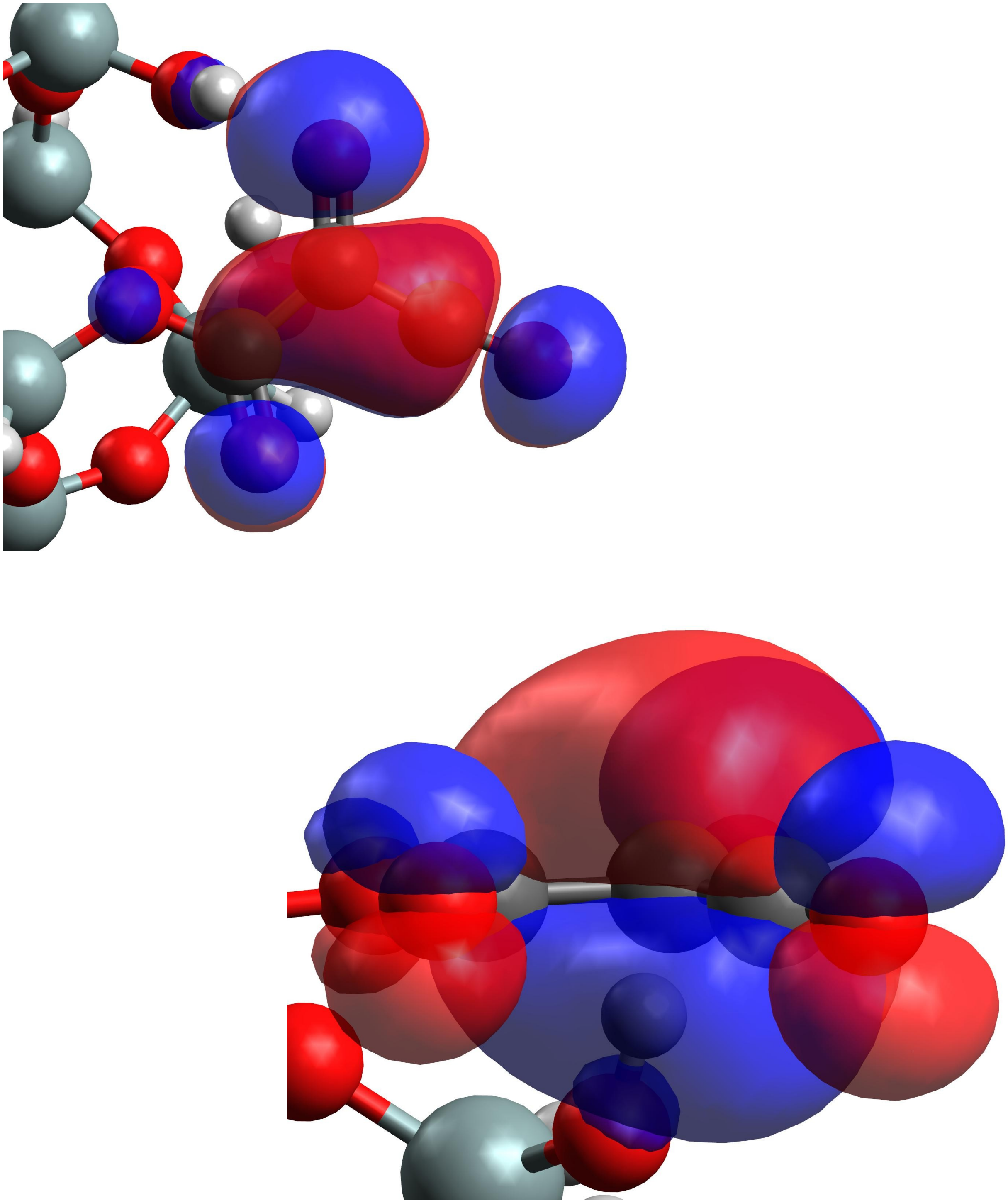

Supplementary Figure S4. SOMO of the $SiO-(CO)_3$ moiety. The radical is partially de-localized on the C atoms.

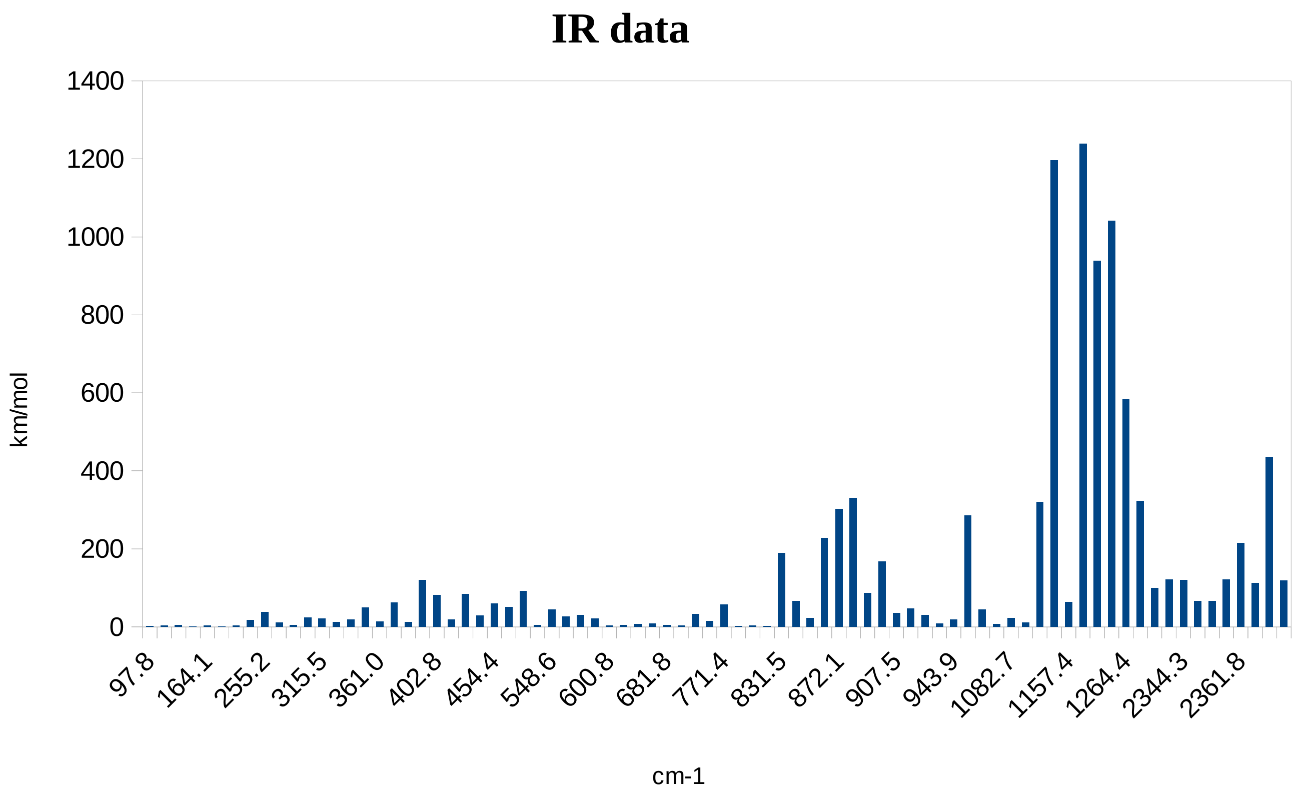

**cm-1**	**I (km/mol)**	**micron**	
**1181.06**	**637.1753**	**8.47**	Si**O-C**O
**1888.38**	**322.8276**	**5.30**	SiO-**CO**

Supplementary Figure S5. Calculated IR spectra of the POSS-CO compound **(2)**.

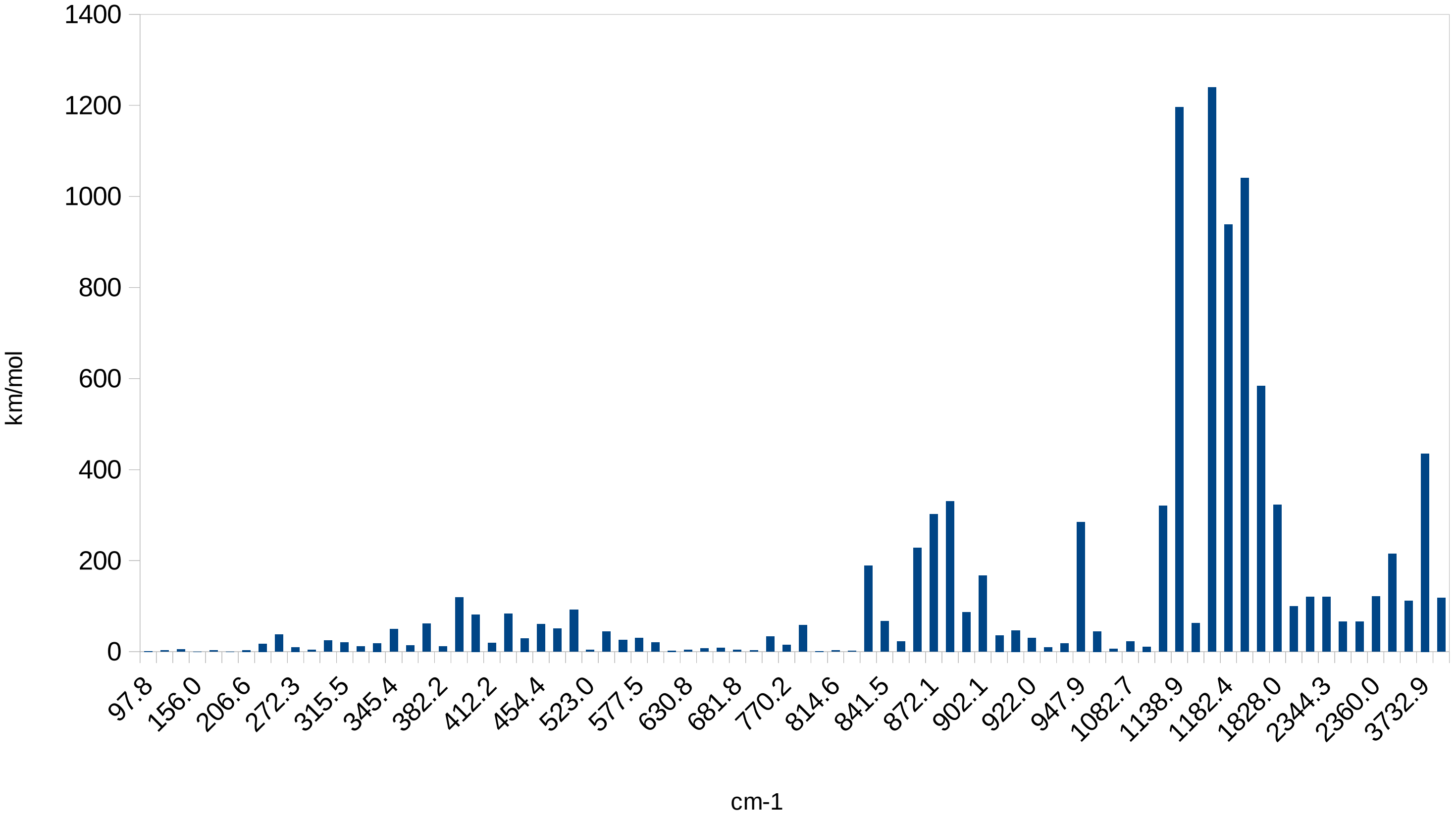

cm-1	I (km/mol)	micron	
**1264.42**	**583.2441**	**7.91**	Si**O-C**O-CO
**1827.96**	**322.6210**	**5.47**	SiO-**CO**-CO
**1997.42**	**99.6875**	**5.01**	SiO-CO-**CO**

Supplementary Figure S6. Calculated IR spectra of the POSS-CO-CO compound **(3)**.

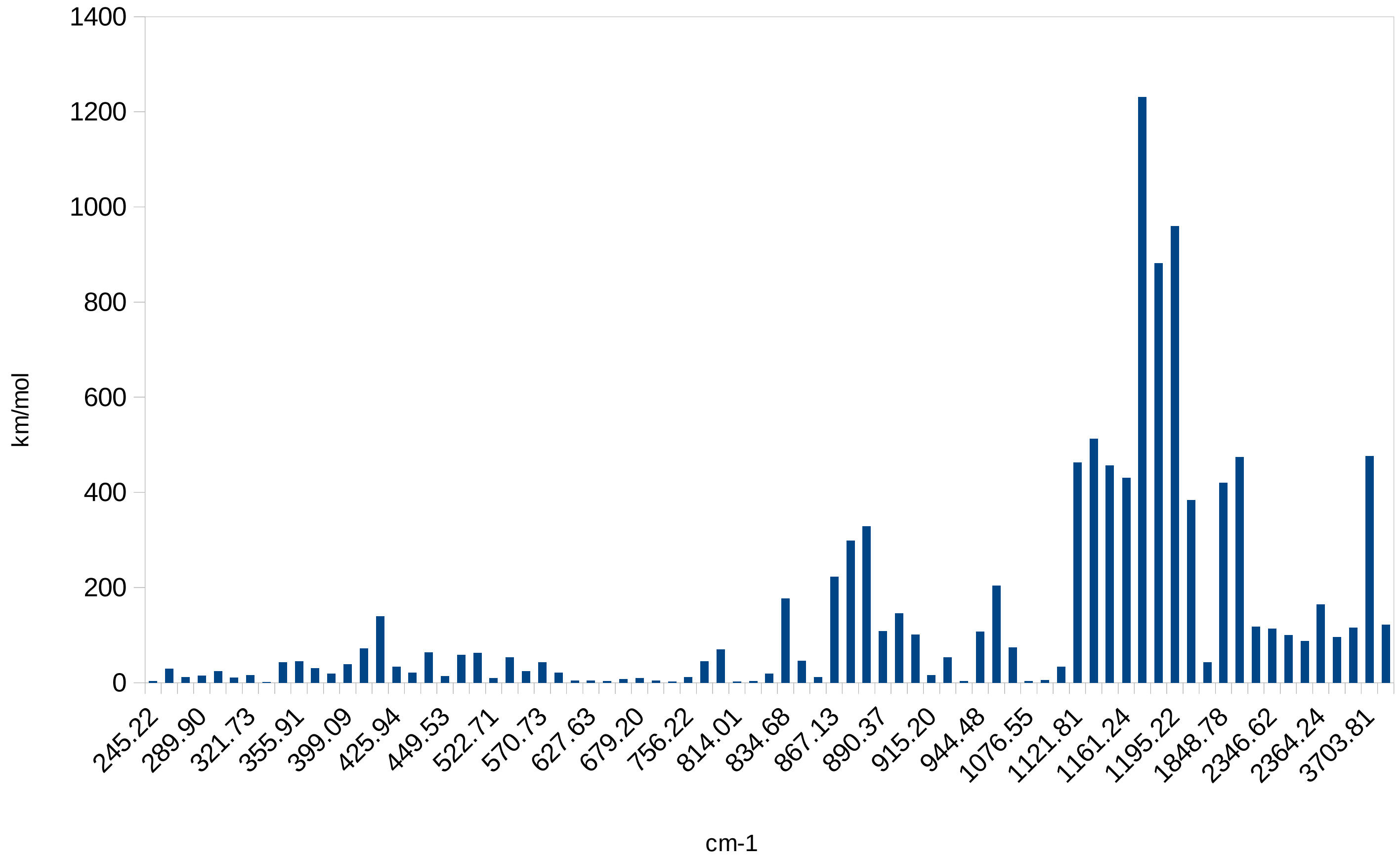

cm-1	I (km/mol)	micron	
**1271.52**	**384.0532**	**7.86**	Si**O-C**O-CO-CO
**1518.72**	**42.6794**	**6.58**	SiO-CO-**CO**-CO
**1848.78**	**419.7911**	**5.41**	SiO-**CO**-CO-CO
**2196.24**	**473.9955**	**4.55**	SiO-CO-CO-**CO**

Supplementary Figure S7. Calculated IR spectra of the POSS-CO-CO-CO compound **(5)**.

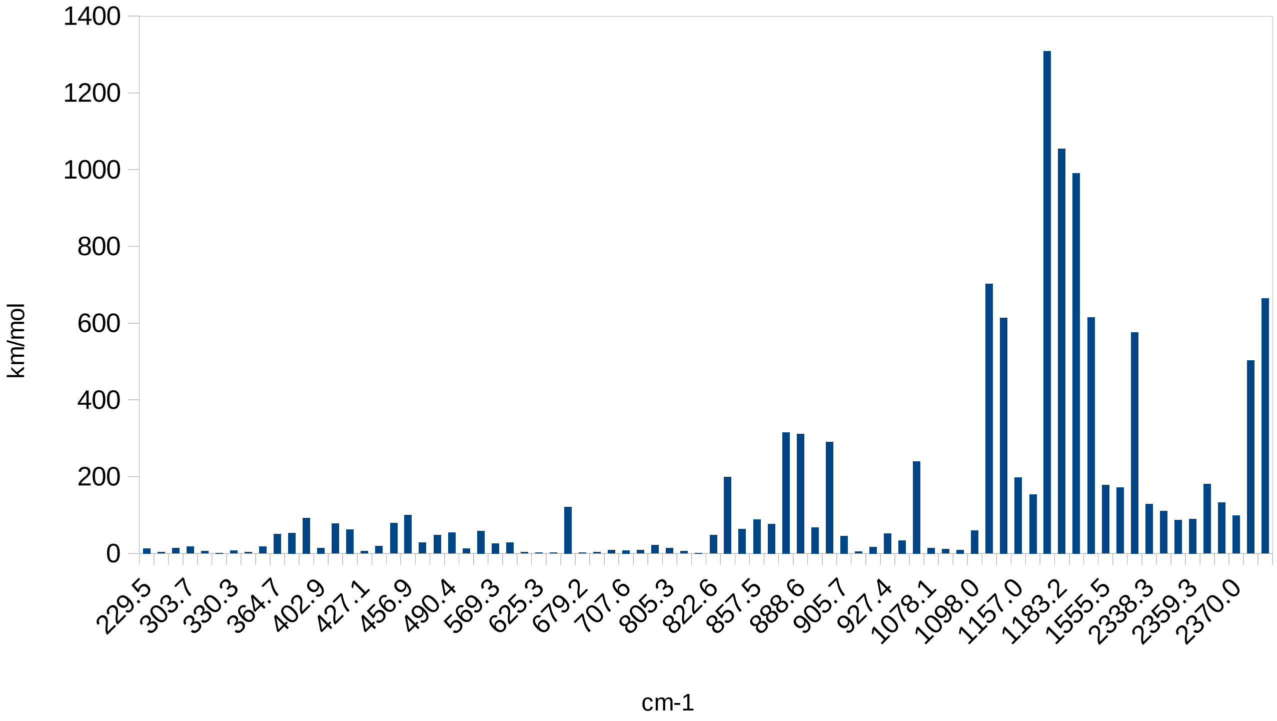

cm-1	I (km/mol)	micron	
**1362.08**	**615.0586**	**7.34**	Si**O-C**O-CO-CO
**1555.54**	**177.925**	**6.43**	SiO-CO-**CO**-CO
**1774.66**	**171.2066**	**5.63**	SiO-**CO**-CO-CO
**2225.81**	**575.976**	**4.49**	SiO-CO-CO-**CO**

Supplementary Figure S8. Calculated IR spectra of the POSS-CO-CO-CO compound **(6)**.

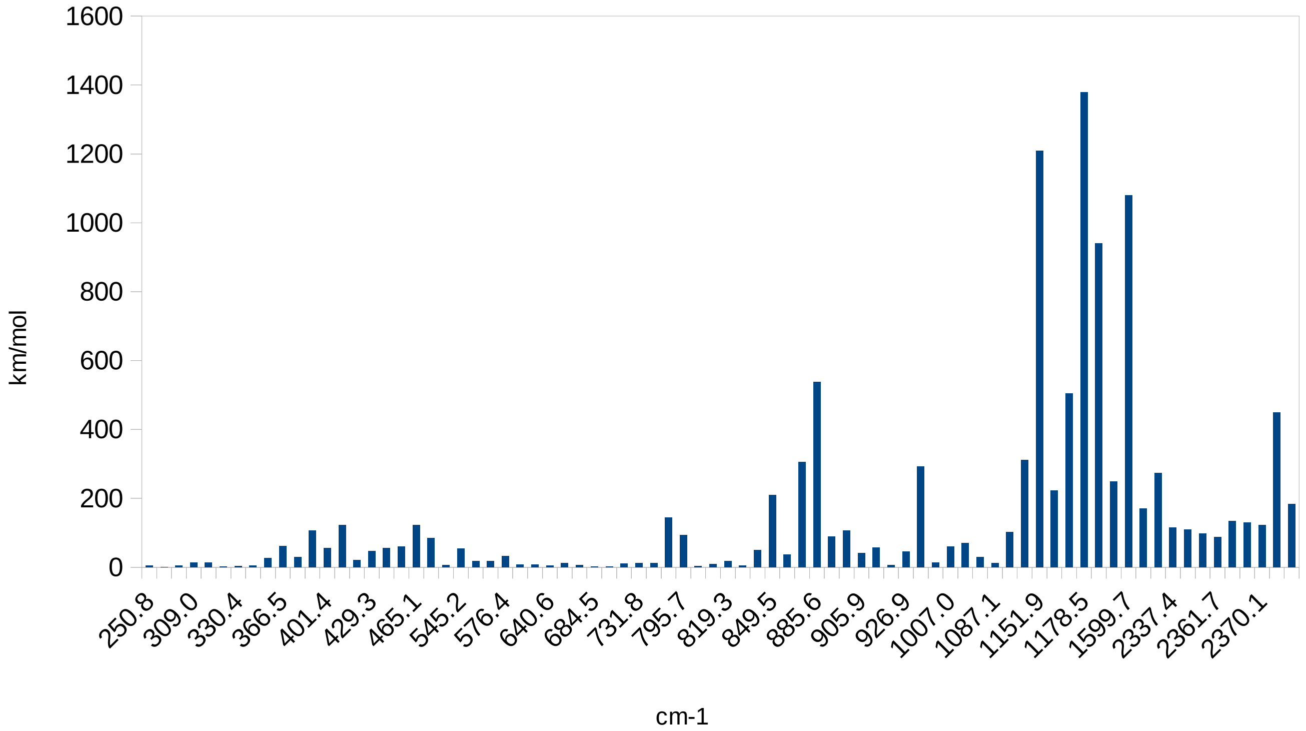

cm-1	I (km/mol)	micron	
**1309.35**	**249.5651**	**7.64**	SiO-**CO-C**O-CO
**1599.7**	**1079.5884**	**6.25**	Si**O-C**O-CO-CO
**1765.87**	**171.0151**	**5.66**	SiO-CO-CO-**CO**
**1979.67**	**273.3433**	**5.05**	SiO-CO-**CO**-CO

Supplementary Figure S9. Calculated IR spectra of the POSS-CO-CO-CO compound **(7)**.

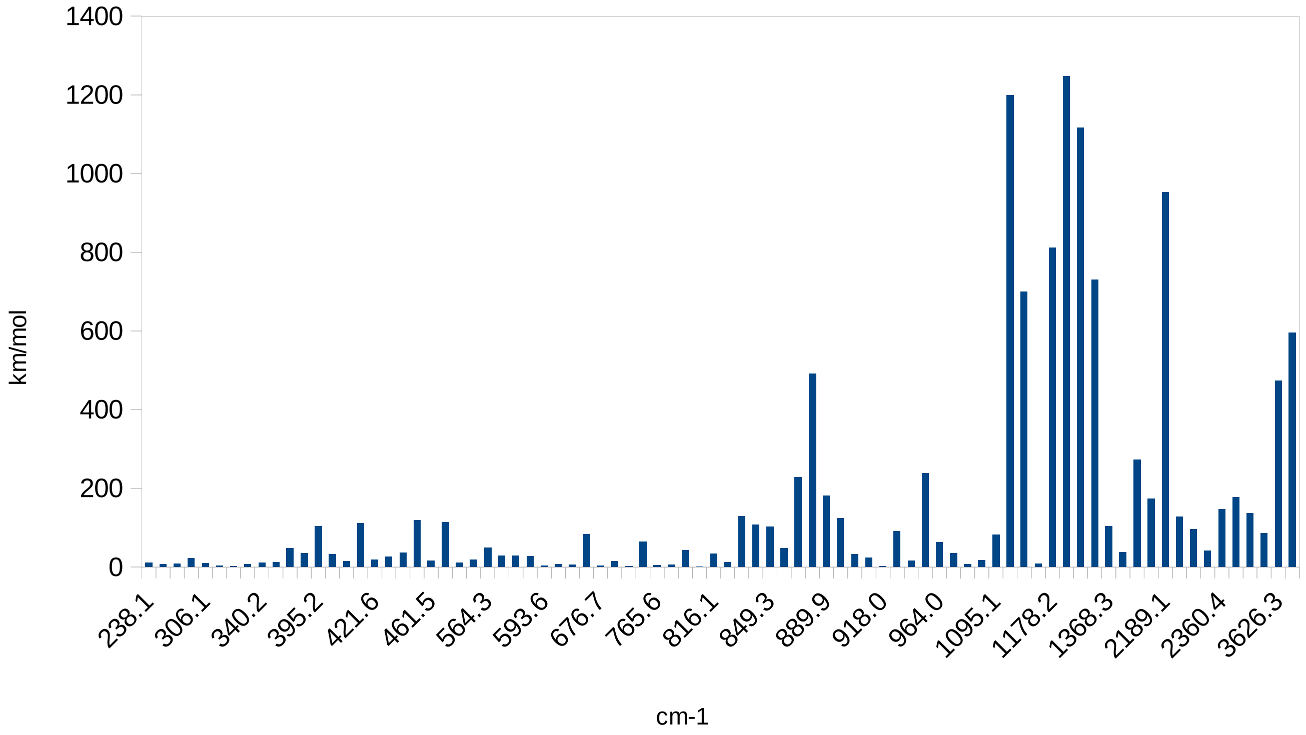

cm-1	I (km/mol)	micron	
**1368.27**	**103.6345**	**7.31**	Si**O-CO**-CO-CO-CO
**1567.49**	**37.9047**	**6.38**	SiO-CO-CO-**CO**-CO
**1689.21**	**272.2286**	**5.92**	SiO-CO-**CO**-CO-CO
**1832.48**	**172.5061**	**5.46**	SiO-**CO**-CO-CO-CO
**2189.11**	**952.3718**	**4.57**	SiO-CO-CO-CO-**CO**

Supplementary Figure S10. Calculated IR spectra of the POSS-CO-CO-CO-CO compound **(8)**.

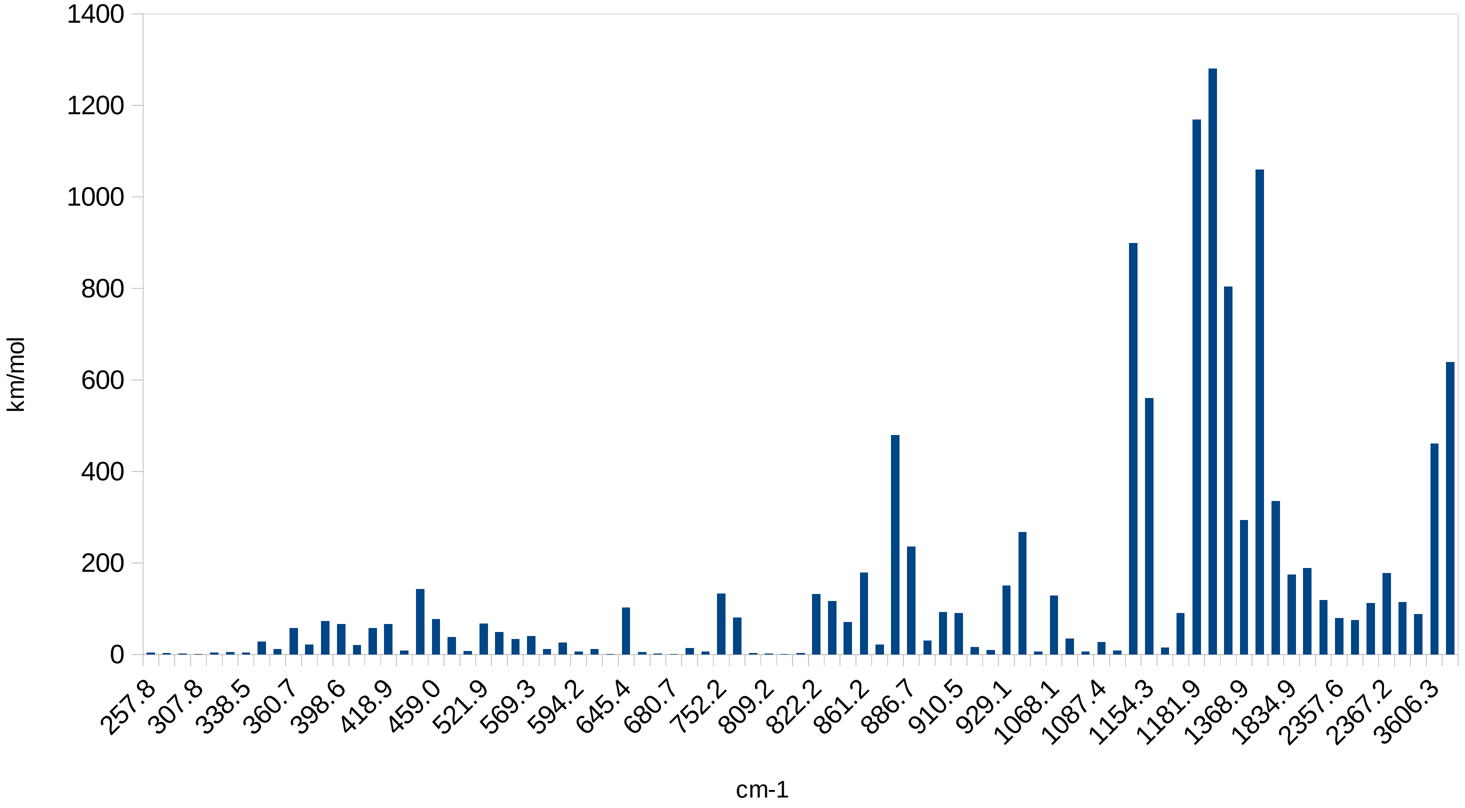

cm-1	I (km/mol)	micron	
**1368.86**	**293.5811**	**7.31**	SiO-**CO**-CO-CO-CO
**1575.53**	**1058.8598**	**6.35**	Si**O-C**O-CO-CO-CO
**1636.71**	**334.7539**	**6.11**	SiO-CO-**CO**-CO-CO
**1834.86**	**174.1066**	**5.45**	SiO-CO-CO-**CO-CO**
**1979.72**	**188.4925**	**5.05**	SiO-CO-CO-CO-**CO**

Supplementary Figure S11. Calculated IR spectra of the POSS-CO-CO-CO-CO compound **(9)**.

2	cm-1	I (km/mol)	micron	
	1181.06	637.1753	8.47	Si**O-C**O
	1888.38	322.8276	5.30	SiO-**CO**

3	cm-1	I (km/mol)	micron	
	1264.42	583.2441	7.91	Si**O-C**O-CO
	1827.96	322.6210	5.47	SiO-**CO**-CO
	1997.42	99.6875	5.01	SiO-CO-**CO**

5	cm-1	I (km/mol)	micron	
	1271.52	384.0532	7.86	Si**O-C**O-CO-CO
	1518.72	42.6794	6.58	SiO-CO-**CO**-CO
	1848.78	419.7911	5.41	SiO-**CO**-CO-CO
	2196.24	473.9955	4.55	SiO-CO-CO-**CO**

6	cm-1	I (km/mol)	micron	
	1362.08	615.0586	7.34	Si**O-C**O-CO-CO
	1555.54	177.925	6.43	SiO-CO-**CO**-CO
	1774.66	171.2066	5.63	SiO-**CO**-CO-CO
	2225.81	575.976	4.49	SiO-CO-CO-**CO**

8	cm-1	I (km/mol)	micron	
	1368.27	103.6345	7.31	Si**O-CO**-CO-CO-CO
	1567.49	37.9047	6.38	SiO-CO-CO-**CO**-CO
	1689.21	272.2286	5.92	SiO-CO-**CO**-CO-CO
	1832.48	172.5061	5.46	SiO-**CO**-CO-CO-CO
	2189.11	952.3718	4.57	SiO-CO-CO-CO-**CO**

Cyclic 7	cm-1	I (km/mol)	micron	
	1309.35	249.5651	7.64	SiO-**CO**-CO-CO
	1599.70	1079.5884	6.25	Si**O-C**O-CO-CO
	1765.87	171.0151	5.66	SiO-CO-CO-**CO**
	1979.67	273.3433	5.05	SiO-CO-**CO**-CO

Cyclic 9	cm-1	I (km/mol)	micron	
	1368.86	293.5811	7.31	SiO-**CO**-CO-CO-CO
	1575.53	1058.8598	6.35	Si**O-C**O-CO-CO-CO
	1636.71	334.7539	6.11	SiO-CO-**CO**-CO-CO
	1834.86	174.1066	5.45	SiO-CO-CO-**CO**-CO
	1979.72	188.4925	5.05	SiO-CO-CO-CO-**CO**

Supplementary Figure S12. Synthesis of the (s) IR lines of the analyzed POSS-$(CO)_n$ compound .

Spin Densities

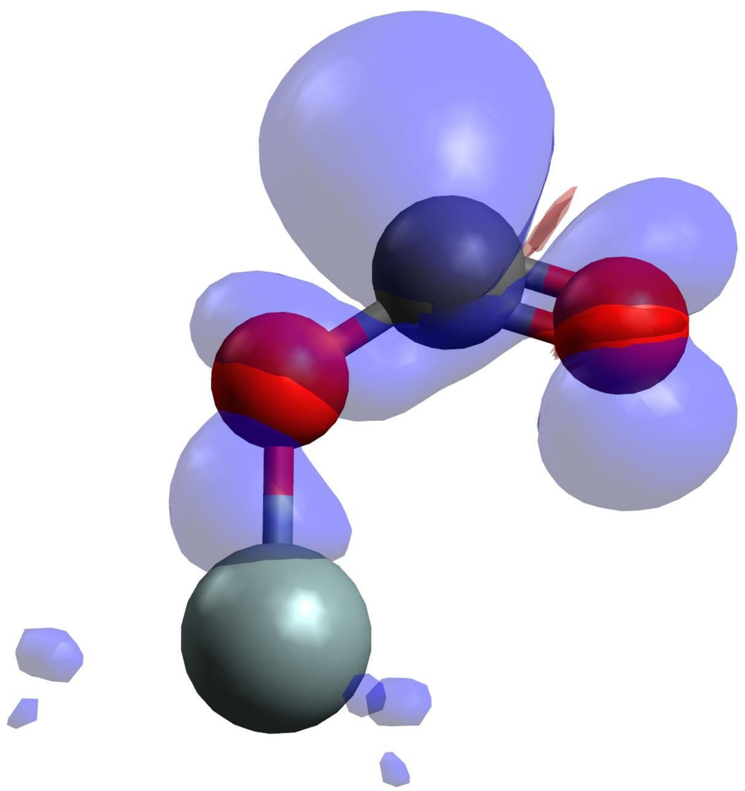

26 O s	:	0.012654	s :	0.012654
pz	:	0.014948	p :	0.180958
px	:	0.071194		
py	:	0.094816		
d :	0.013019			
27 C s	:	0.171971	s :	0.171971
pz	:	0.091186	p :	0.370926
px	:	0.041778		
py	:	0.237963		
d :	0.098130			

26 O	0.20785
27 C	0.64506

Supplementary Figure S13. Spin densities (e/bohr3) of the SiO-CO. Spin values in the Table Loewdin analysis on reduced orbitals.

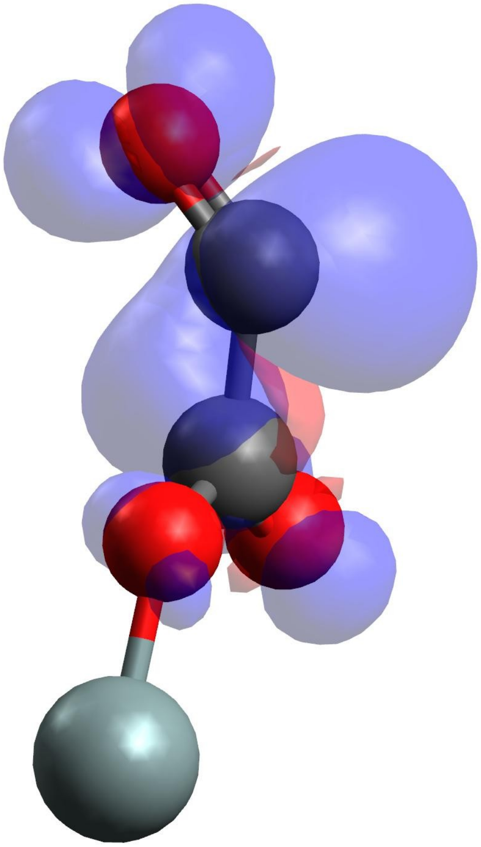

26 O :	0.066946
27 C :	0.127920
30 C :	0.523285
31 O :	0.257461

26 O s	:	0.001027	s :	0.001027
pz	:	-0.001663	p :	0.063308
px	:	-0.002520		
py	:	0.067491		
d :		0.002369		
27 C s	:	0.016718	s :	0.016718
pz	:	0.006457	p :	0.073770
px	:	0.003238		
py	:	0.064075		
d :		0.033827		
30 C s	:	0.077624	s :	0.077624
pz	:	0.245179	p :	0.351600
px	:	0.056390		
py	:	0.050031		
d :		0.083248		
31 O s	:	0.006513	s :	0.006513
pz	:	0.192351	p :	0.230344
px	:	0.031642		
py	:	0.006352		
d :		0.018955		

Supplementary Figure S14. Spin densities (e/bohr3) of the SiO-CO-CO. Spin values in the Table Loewdin analysis on reduced orbitals.

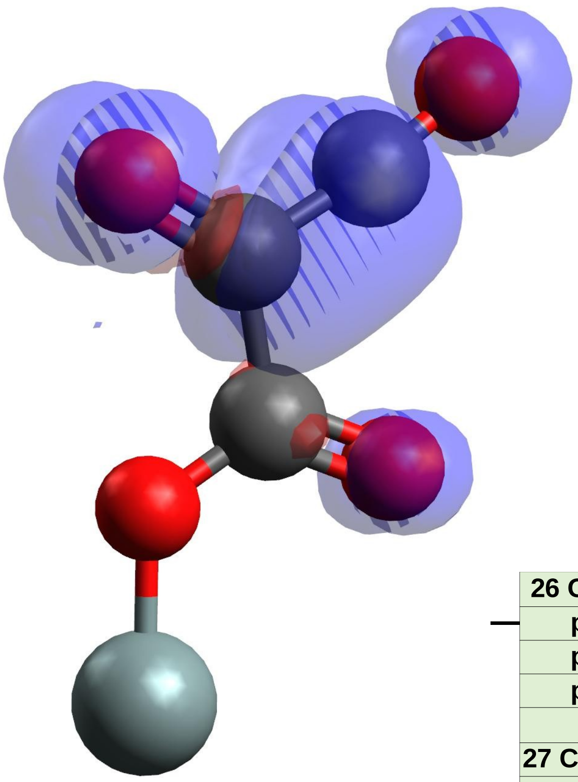

26 O :	0.062711
27 C :	0.015682
30 C :	0.188007
31 O :	0.370969
32 C :	0.217303
33 O :	0.140568

26 O s	:	0.000854	s :	0.000854
pz	:	0.038480	p :	0.061961
px	:	0.003012		
py	:	0.020470		
d :		-0.000059		
27 C s	:	-0.001763	s :	-0.001763
pz	:	-0.003448	p :	-0.008319
px	:	-0.000643		
py	:	-0.004228		
d :		0.022151		
30 C s	:	0.000522	s :	0.000522
pz	:	0.058887	p :	0.105121
px	:	0.003238		
py	:	0.042996		
d :		0.070251		
31 O s	:	0.005477	s :	0.005477
pz	:	0.201098	p :	0.359410
px	:	0.017272		
py	:	0.141040		
d :		0.005632		
32 C s	:	0.001766	s :	0.001766
pz	:	0.087436	p :	0.153949
px	:	0.010459		
py	:	0.056054		
d :		0.059101		

Supplementary Figure S15. Spin densities (e/bohr3) of the SiO-CO-CO-CO. Spin values in the Table Loewdin analysis on reduced orbitals.

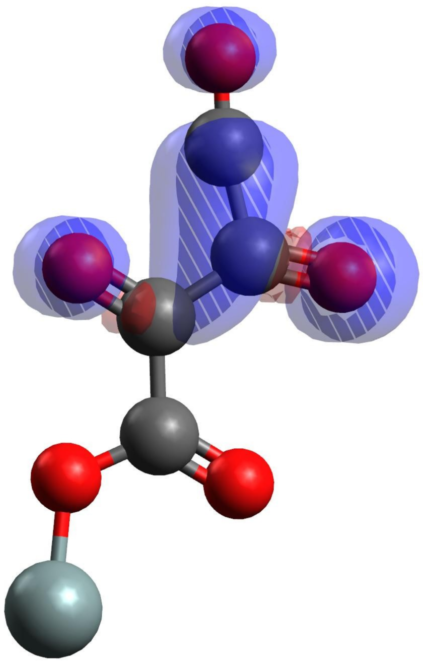

26 O s	:	0.000012	s :	0.000012
pz	:	0.002794	p :	0.003740
px	:	0.000031		
py	:	0.000915		
d :		0.000029		
27 C s	:	-0.000265	s :	-0.000265
pz	:	0.000187	p :	-0.000173
px	:	-0.000072		
py	:	-0.000288		
d :		0.000750		
30 C s	:	-0.001328	s :	-0.001328
pz	:	0.004823	p :	0.005451
px	:	-0.002582		
py	:	0.003210		
d :		0.028653		
31 O s	:	0.002107	s :	0.002107
pz	:	0.079057	p :	0.135438
px	:	0.006619		
py	:	0.049763		
d :		0.001024		
32 C s	:	-0.000620	s :	-0.000620
pz	:	0.044206	p :	0.067791
px	:	0.000465		
py	:	0.023120		
d :		0.069733		
33 O s	:	0.005330	s :	0.005330
pz	:	0.242577	p :	0.397966
px	:	0.019268		
py	:	0.136121		
d :		0.004132		
34 O s	:	0.001261	s :	0.001261
pz	:	0.056942	p :	0.089143
px	:	0.001905		
py	:	0.030297		
d :		0.007783		

26 O :	0.003783
27 C :	0.000476
30 C :	0.038804
31 O :	0.138599
32 C :	0.149590
33 O :	0.407723
34 O :	0.098911
35 C :	0.161214

Supplementary Figure S16. Spin densities (e/bohr3) of the SiO-CO-CO-CO-CO. Spin values in the Table Loewdin analysis on reduced orbitals.

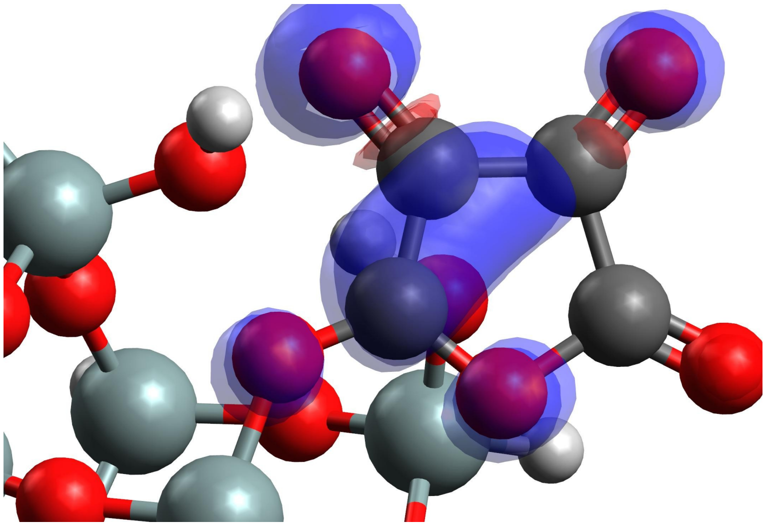

Supplementary Figure S17. Spin densities (e/bohr3) of the SiO-CO-CO-CO-CO. Spin values in the Table Loewdin analysis on reduced orbitals.

26 O :	0.093472
27 C :	0.212824
30 C :	0.149821
31 O :	0.341776
32 C :	0.031238
33 O :	0.098362
34 O :	-0.014551
35 C :	0.005962

26 O s	:	0.002034	s :	0.002034
pz	:	0.034372	p :	0.084308
px	:	0.014529		
py	:	0.035408		
d :	0.006644			
27 C s	:	0.003855	s :	0.003855
pz	:	0.072365	p :	0.175855
px	:	0.024199		
py	:	0.079291		
d :	0.029588			
30 C s	:	-0.000804	s :	-0.000804
pz	:	0.029262	p :	0.067852
px	:	0.008894		
py	:	0.029695		
d :	0.073889			
31 O s	:	0.005004	s :	0.005004
pz	:	0.157560	p :	0.332988
px	:	0.032542		
py	:	0.142886		
d :	0.003550			
32 C s	:	-0.001305	s :	-0.001305
pz	:	-0.001822	p :	-0.001955
px	:	-0.000630		
py	:	0.000497		
d :	0.031786			
33 O s	:	0.001234	s :	0.001234
pz	:	0.032564	p :	0.097220
px	:	0.015814		
py	:	0.048841		
dz2	:	-0.000072	d :	-0.000048
34 O s	:	-0.000207	s :	-0.000207
pz	:	-0.004883	p :	-0.014454
px	:	-0.002768		
py	:	-0.006802		
d :	0.000092			
C s	:	-0.000341	s :	-0.000341
pz	:	0.001667	p :	0.001908
px	:	-0.000209		
py	:	0.000450		
d :	0.003364			

PES first Hydrogenation

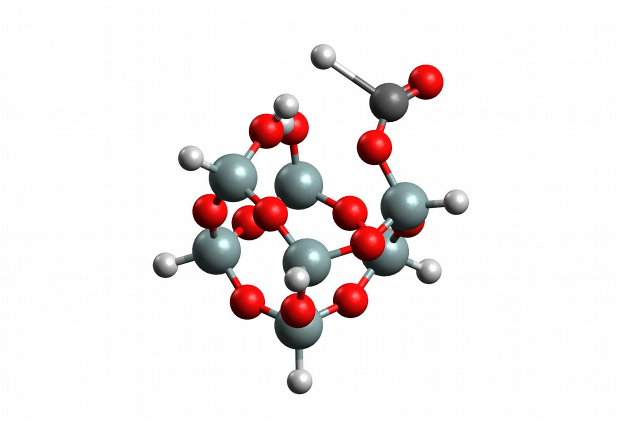

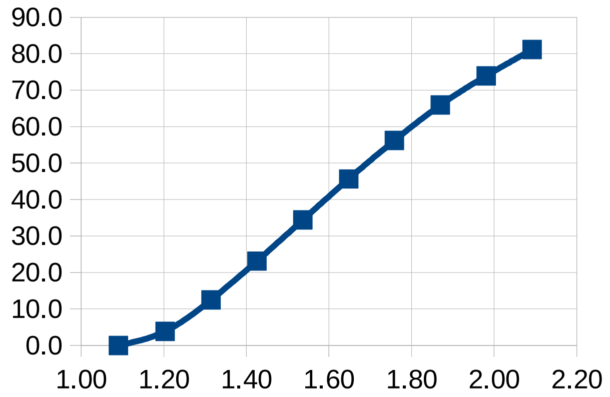

Supplementary Figure S18. Hydrogenation PES of the reaction: SiO-CO+H→ SiO-CHO

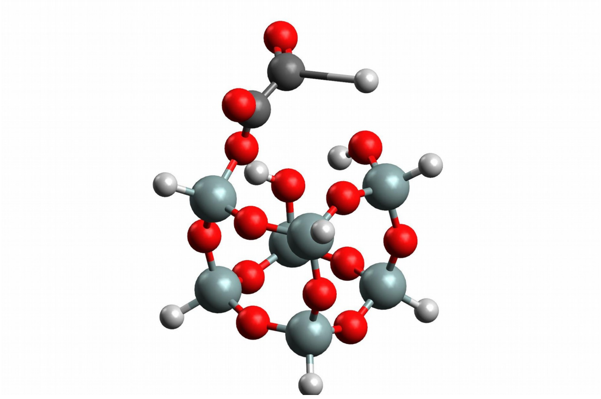

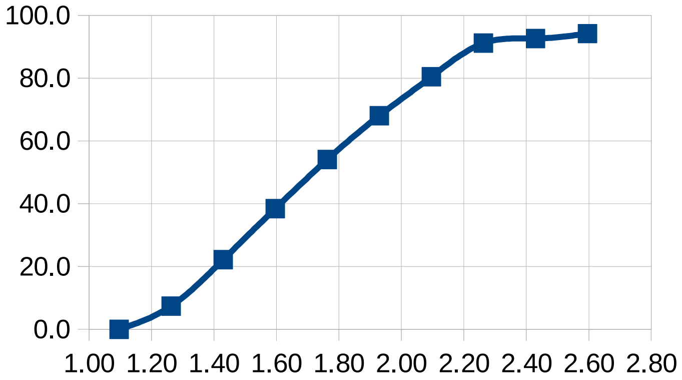

Supplementary Figure S19. Hydrogenation PES of the reaction: SiO-CO-CO+H→ SiO-CO-CHO

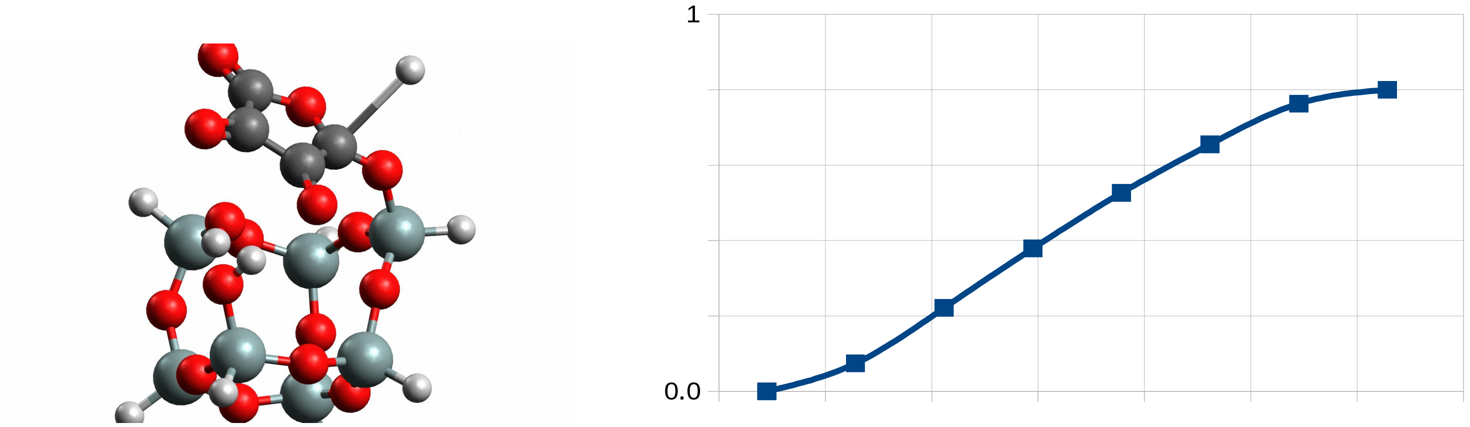

Supplementary Figure S20. Hydrogenation PES of the reaction: SiO-$(CO)_4$ (ring)+H→ SiO-CHO-$(CO)_3$ (ring)

Supplementary Material

Figure 7 XYZ

Compound 10

M=2

Si	-2.99965427519386	1.72874095689677	2.96074042760227
O	-2.12414389364120	0.41840175190998	2.37497758195146
O	-3.27690941218515	-2.54393058387706	2.37530664741589
O	-4.51162111353807	1.38955092068540	2.52311663730342
Si	-4.89527342583870	-2.53452009933293	2.58897391842839
O	-5.49946993365845	-1.05434099921693	2.31361009018719
Si	-5.82265433420386	0.46092303425551	2.78009741371486
O	-5.12938603098661	-2.97811190199779	4.12537515709656
H	-5.56896219445179	-3.47442753391219	1.70112947549417
H	-6.92812295452474	0.98930304690950	1.99575166861054
O	-6.20519790676257	0.48502158954220	4.35085397856046
O	-1.38200294055174	-2.00487419440291	4.35738255261261
H	-2.55335790969409	2.95661033051630	2.32456521440780
O	-2.84082419648138	1.80861110894788	4.55403202460226
O	-3.32653424225480	-2.65845860596758	6.04669487790624
Si	-4.90795757485082	-2.74870996999575	5.71681306898796
Si	-1.95561517115074	-1.79964080016951	5.86100316269415
O	-5.64102142924497	-1.36965926959440	6.15550702437740
O	-2.28115819154804	-0.23207432450183	6.14082363040392
O	-4.58928860760457	1.04837542375089	6.37097541224722
Si	-5.90341429976878	0.21004547744513	5.92221097314608
Si	-3.00471996404431	1.20453939030470	6.05542912868215
H	-0.95967473161409	-2.23639948536526	6.82983602922994

H	-2.41357269575269	2.13168928865199	7.00716991781757
H	-7.04525808333330	0.63901102043556	6.71506323837993
H	-5.49336004557490	-3.86338105564196	6.44600734531144
O	-0.12800849660072	1.11417384240379	3.12129355786348
C	-0.81679366720237	0.31649272022900	2.55410872773529
H	-2.83812888028395	-2.08996621652856	1.65128140284245
H	-2.00781247333587	-2.31251657628785	3.68470320884478
C	-0.21204356141898	-0.92025157903077	1.91004690508555
O	-0.82823764271952	-1.65232134157272	1.19448047281943
H	0.85212480314878	-1.05025908594466	2.13722435479080
H	-0.43495452313311	3.63269372045608	0.52531477284631

TS V

M=2

Si	-3.11219081344685	1.85643415051743	3.07009591720579
O	-2.14892508345425	0.66573306096977	2.38814211014030
O	-3.27342716186623	-2.30110889500801	2.35123102257266
O	-4.59385159100977	1.41042320014217	2.62958162828128
Si	-4.88810052515420	-2.44965509213250	2.53314266543310
O	-5.62015629731069	-1.01635051562999	2.32230019160910
Si	-5.91337139089957	0.48551948978626	2.85313554755266
O	-5.10294742898159	-2.98986619558102	4.04125693894713
H	-5.46627995849893	-3.39518267959833	1.58623834767486
H	-7.01640690297792	1.05697477050321	2.09587143003025
O	-6.28726732221051	0.45570757048013	4.42595939526605

Supplementary Material

O	-1.32315387015541	-1.84561100872853	4.30067496535078
H	-2.77202892811351	3.14861784280932	2.49433591662684
O	-2.93242354118873	1.87470252443828	4.66253104868311
O	-3.26851750073681	-2.64621923649298	5.92809995073501
Si	-4.85082179237801	-2.79425404818088	5.63340528378624
Si	-1.91924342390755	-1.74202056855722	5.80457971450937
O	-5.63391095036009	-1.46170316757677	6.12592634025862
O	-2.30408349999710	-0.20730593176938	6.17443663945716
O	-4.65426929291998	0.97978351094386	6.44151220183503
Si	-5.94730716993031	0.11856160027060	5.97724210356497
Si	-3.07543548321250	1.20502554833193	6.13853242414462
H	-0.91948741813333	-2.19900884339531	6.76036903925594
H	-2.52023762073829	2.11838504138563	7.12509150143506
H	-7.08627048287666	0.47504175759447	6.80931995058538
H	-5.37247695168842	-3.95129021816550	6.34461254540192
O	-0.17530334655237	1.32468580855582	3.23073140748057
C	-0.83764533501854	0.59486670191312	2.55359432806194
H	-2.85577079548348	-1.81754024133678	1.63355682637843
H	-1.94624653521500	-2.11595606285790	3.61051738557669
C	-0.21300886243654	-0.54915349129264	1.76240844648912
O	-0.86601199769555	-1.33406880019410	1.11890783808369
H	0.85988665830496	-0.67358567979773	1.95097571111833
H	0.36368261624381	0.72975809765357	0.49358323646797

Compound 11

M=2

Si	-3.13252587028838	1.94559647803817	3.10252443113222
O	-2.26260090336721	0.69364505502566	2.38407666932125
O	-3.15721638855639	-2.33564178262756	2.26917939844385
O	-4.65843724362997	1.58078425390824	2.73833770171092
Si	-4.77238954616676	-2.38703753678782	2.49206869447811
O	-5.42221471054340	-0.91341087785507	2.29931596974782
Si	-5.88689623034742	0.52572349811374	2.87669363667539
O	-4.98884330794566	-2.91702520100647	4.00358682780068
H	-5.42098740888376	-3.29989582712187	1.55839996005592
H	-7.01769573178745	1.02211155491912	2.10738964269559
O	-6.30117937566167	0.39378314093712	4.43475313841797
O	-1.30225484842462	-1.90972367742995	4.29517332583377
H	-2.76931328310838	3.22575726942780	2.51914747629625
O	-2.87474023695807	1.92876525301782	4.68827116608716
O	-3.23552290740878	-2.62326331306760	5.97154585157721
Si	-4.80278728833958	-2.79637705138233	5.61047729389082
Si	-1.87544300607313	-1.74573095527145	5.80405273543888
O	-5.63432521042982	-1.50819198887379	6.14026990493190
O	-2.21828281534706	-0.19084459514561	6.12792765932943
O	-4.60726891687299	0.91990937600507	6.40439425396237
Si	-5.92551525678004	0.07611810114089	5.98092894100303
Si	-3.03282731642163	1.19947033110473	6.13380073560416
H	-0.86828040140945	-2.19484841122408	6.75554570781678

H	-2.51590773227318	2.08744738384280	7.16356505588269
H	-7.03874486448206	0.45863304437476	6.83581964736840
H	-5.31917935554560	-3.99815461477253	6.24709332501730
O	-0.29434291578057	1.66634240565191	2.78529710559520
C	-0.93987223791546	0.76680153020114	2.33446409587993
H	-2.73859846080505	-1.79057588304840	1.59170849257327
H	-1.94244337925436	-2.15366279890547	3.60837790731542
C	-0.27968481730308	-0.41544640741697	1.63111335636147
O	-1.07198490879255	-1.12566159719024	0.81673147664753
H	-0.02519245911024	-1.08634105297573	2.47688710887480
H	0.66048933601386	-0.08871510360604	1.18298130623244

TS VI

M=2

Si	-3.07169531671783	1.98704582134097	3.06826188315253
O	-2.11662438144939	0.92471511723240	2.19942999125623
O	-3.05153859928520	-2.29800462066691	2.62868769152903
O	-4.56262064202075	1.55009322064538	2.65583303213504
Si	-4.67882535199137	-2.40437324447750	2.63164783088950
O	-5.34007069530499	-0.95263219196273	2.32859779683071
Si	-5.80729606284460	0.51639268483830	2.81902789635360
O	-5.08046317701384	-2.93092045929257	4.10499871784443
H	-5.17702208225664	-3.34195648596797	1.63128512959783
H	-6.91116951297050	0.98402811562514	1.99442840592709

Supplementary Material

O	-6.26703562150573	0.47490164370907	4.36767906878399
O	-1.36030653763520	-2.78400650071571	4.83626113399458
H	-2.79748797659871	3.35236338836810	2.64736007875948
O	-2.80653979110307	1.81406634033223	4.64060433460935
O	-3.60912720690435	-2.59923364083681	6.29381539900006
Si	-5.10800829380686	-2.78626260725391	5.72383364773579
Si	-2.08667741181723	-2.05591405022166	6.07898955777958
O	-5.99617800039404	-1.48293272772807	6.12496559934185
O	-2.17101598106534	-0.44075705383579	5.83270361534051
O	-4.51144760485535	0.70707766902907	6.33386348586940
Si	-5.96874710334695	0.12502747751945	5.92540138186337
Si	-2.94149511331058	0.96114724250360	6.02171273324111
H	-1.31138709194141	-2.32914788817346	7.28275686323519
H	-2.36102625187424	1.74488468474093	7.10289689013786
H	-6.99177153029033	0.73302453228907	6.76284614218271
H	-5.72464079327629	-3.97206297863582	6.30013215263347
O	-0.07473410244949	1.86881305193867	2.57312838554983
C	-0.79781294491807	1.04253927833276	2.03928802920894
H	-2.55402102192677	-1.82423114367349	1.95502506924676
H	-1.82470191067721	-2.75452577291819	3.99046512523146
C	-0.16964092401766	-0.24545537222943	1.40370639526041
O	-0.82760195808310	-1.22846672814380	1.15556623889830
H	0.90973334173851	-0.14081107277933	1.30904448934126
H	-0.60524234808540	0.96362427106803	0.74608580723872

Supplementary Material

Compound 12

M=2

Si	-2.95819883786238	1.76606036386446	3.04691622210578
O	-2.15500754391560	0.96762501130512	1.83883027312500
O	-3.08246020605414	-1.92224430745786	2.90898216256570
O	-4.51599648968982	1.44972915556800	2.77430015652544
Si	-4.65658453229135	-2.29793112741535	2.66635081670554
O	-5.48913325040194	-0.96412208762051	2.25572351833584
Si	-5.84316844060901	0.51790118365021	2.81732484015140
O	-5.17459919073784	-2.89816558642995	4.07294569524752
H	-4.84341193956514	-3.28558965816732	1.60944418884399
H	-6.87402687530864	1.11368453787864	1.98078435253874
O	-6.37601312550150	0.41005014244212	4.34230039911065
O	-1.34878604356896	-2.83216799383594	5.00231767702552
H	-2.72210324421847	3.20065851263226	2.93574632673582
O	-2.48960913946542	1.21558387478192	4.48670874969827
O	-3.70213241730020	-2.96371376346658	6.28675156974536
Si	-5.20067527018152	-2.84314255594491	5.69878510728666
Si	-2.19850579863873	-2.32922141391715	6.28098984924407
O	-5.86606766815996	-1.43727458445076	6.16971330229888
O	-2.34288392712843	-0.70776397284440	6.29104706691791
O	-4.49824053372671	0.83486207760351	6.15421017496593
Si	-5.94471311388934	0.15469745331805	5.88800760564644
Si	-2.88311581462908	0.77577747427248	6.00204477481136
H	-1.49615896529913	-2.75497992191762	7.48449044964172

Supplementary Material

H	-2.28263922692142	1.71358968251469	6.94153855623896
H	-6.93519110506236	0.75591029710373	6.76864934731298
H	-6.01187593428354	-3.94370217670644	6.19781550605406
O	-0.12082436477577	1.95113224236039	2.27971411796066
C	-0.84907550681391	1.22889528880179	1.49064381851267
H	-2.57676130238961	-1.42073606841409	2.26466521595893
H	-1.75467358658289	-2.66186362306861	4.14594136239395
C	-0.05968504108278	-0.15665440934292	1.32253011875275
O	-0.59270369768168	-1.19437035672176	1.49125226435520
H	0.98754546242759	-0.01733456203347	1.03494050572990
H	-0.81553332869025	1.58516087165829	0.43949390745637
